# KRAS induces lung tumorigenesis through microRNAs modulation

**DOI:** 10.1038/s41419-017-0243-9

**Published:** 2018-02-13

**Authors:** Lei Shi, Justin Middleton, Young-Jun Jeon, Peter Magee, Dario Veneziano, Alessandro Laganà, Hui-Sun Leong, Sudhakar Sahoo, Matteo Fassan, Richard Booton, Rajesh Shah, Philip A. J. Crosbie, Michela Garofalo

**Affiliations:** 1 Transcriptional Networks in Lung Cancer Group, Cancer Research UK Manchester Institute, The University of Manchester, Wilmslow Road, Manchester, M20 4BX UK; 2 Cancer Research UK Lung Cancer Centre of Excellence, Manchester and University College London, London, UK; 3 Department of Cancer Biology and Genetics, Comprehensive Cancer Center, The Ohio State University, Columbus, OH 43210 USA; 4 Department of Genetics and Genomic Sciences, Icahn School of Medicine at Mount Sinai, New York City, 10029 USA; 5 RNA Biology Group, Cancer Research UK Manchester Institute, The University of Manchester, Wilmslow Road, Manchester, M20 4BX UK; 6 Department of Medicine, Surgical Pathology & Cytopathology Unit, University of Padua, Padua, Italy; 7 Manchester Thoracic Oncology Centre, University Hospital of South Manchester, Southmoor Road, Wythenshawe, M23 9LT UK; 8 Department of Thoracic Surgery, University Hospital of South Manchester, Southmoor Road, Wythenshawe, M23 9LT UK

## Abstract

Oncogenic *KRAS* induces tumor onset and development by modulating gene expression via different molecular mechanisms. MicroRNAs (miRNAs) are small non-coding RNAs that have been established as main players in tumorigenesis. By overexpressing wild type or mutant KRAS (KRAS^G12D^) and using inducible human and mouse cell lines, we analyzed KRAS-regulated microRNAs in non-small-cell lung cancer (NSCLC). We show that miR-30c and miR-21 are significantly upregulated by both KRAS isoforms and induce drug resistance and enhance cell migration/invasion via inhibiting crucial tumor suppressor genes, such as NF1, RASA1, BID, and RASSF8. MiR-30c and miR-21 levels were significantly elevated in tumors from patients that underwent surgical resection of early stages NSCLC compared to normal lung and in plasma from the same patients. Systemic delivery of LNA-anti-miR-21 in combination with cisplatin in vivo completely suppressed the development of lung tumors in a mouse model of lung cancer. Mechanistically, we demonstrated that ELK1 is responsible for miR-30c and miR-21 transcriptional activation by direct binding to the miRNA proximal promoter regions. In summary, our study defines that miR-30c and miR-21 may be valid biomarkers for early NSCLC detection and their silencing could be beneficial for therapeutic applications.

## Introduction

Lung cancer is the primary cause of cancer-related morbidity and mortality worldwide responsible for 1,590,000 deaths in 2012^1^. It is roughly divided into small-cell lung cancer (SCLC) and NSCLC. The latter includes squamous cell carcinoma and adenocarcinoma and represents ~85% of all lung cancer cases. The proto-oncongene *RAS* encodes three different RAS proteins: *HRAS*, *NRAS*, and *KRAS*, regulated by guanine nucleotide exchange factors, which stimulate RAS activation through GDP for GTP exchange, and by GTPase-activating proteins (GAPs), which catalyze the hydrolysis of GTP to GDP to switch off the KRAS signaling^2^. Mutations in KRAS, such as the activating point mutation G12D, lead to constitutive activation of KRAS and, accordingly, of downstream effector pathways, including the RAF/mitogen-activated protein kinase (MAPK)/ERK and phosphoinositide 3-kinase (PI3K)/AKT/mammalian target of rapamycin (mTOR) signalings^2–4^. KRAS mutations confer chemoresistance to drugs such as gefitinib and cisplatin, denoting that inhibition of KRAS might sensitize NSCLC cells to chemotherapeutic agents^5,6^. Although several small molecules have been developed and tested, an effective and specific inhibitor for KRAS remains largely elusive for the clinical treatment of KRAS-driven NSCLC. Targeting downstream proteins of KRAS has been shown to be an effective strategy^7^. The MEK inhibitor selumetinib revealed some clinical benefits combined with docetaxel in metastatic NSCLC patients. However, the side effects have been higher than with docetaxel alone^8^. MicroRNAs are non-coding RNAs that silence gene expression by binding to 3′ untranslated region (3′UTR) of mRNAs, resulting in mRNA decay or translational repression^9^. MiRNAs play vital roles in various pathological processes such as immunity, proliferation and stem cell maintenance^10–12^. Over the past decade, an increasing number of studies reported that miRNAs are closely associated with the etiology of various human cancers^13,14^ acting as oncogenes or tumor suppressors and regulate tumor metastasis^15,16^. A substantial amount of miRNAs are released into the bloodstream where they are highly stable and miRNA profile has been found deranged in the blood of patients compared with that of healthy subjects^17^. Therefore, microRNAs are potential diagnostic and prognostic biomarkers. In this study we analyzed KRAS-modulated miRNAs and their role(s) in NSCLC. Overexpression of KRAS^WT^ or KRAS^G12D^ induced a significant upregulation of miR-30c and miR-21. MiR-30c and miR-21 enhanced cell proliferation and migration/invasion and inhibited apoptosis by targeting important tumor suppressor genes, inducing the activation of KRAS downstream pathways. In addition, we showed that ELK1, a transcription factor downstream of KRAS, directly regulated the expression of miR-30c and miR-21 by binding to the miRNA proximal promoter regions. In NSCLC specimens, miR-30c and miR-21 positively correlated with KRAS^WT^ or KRAS^G12D^ and ELK1 expression. Notably, miR-30c and miR-21 were found highly expressed in matched normal/tumor samples and in the blood of patients that underwent surgical resection of early NSCLC, indicating that they may be useful biomarkers for lung cancer early detection.

## Results

### KRAS-modulated microRNAs

**Fig. 1:**
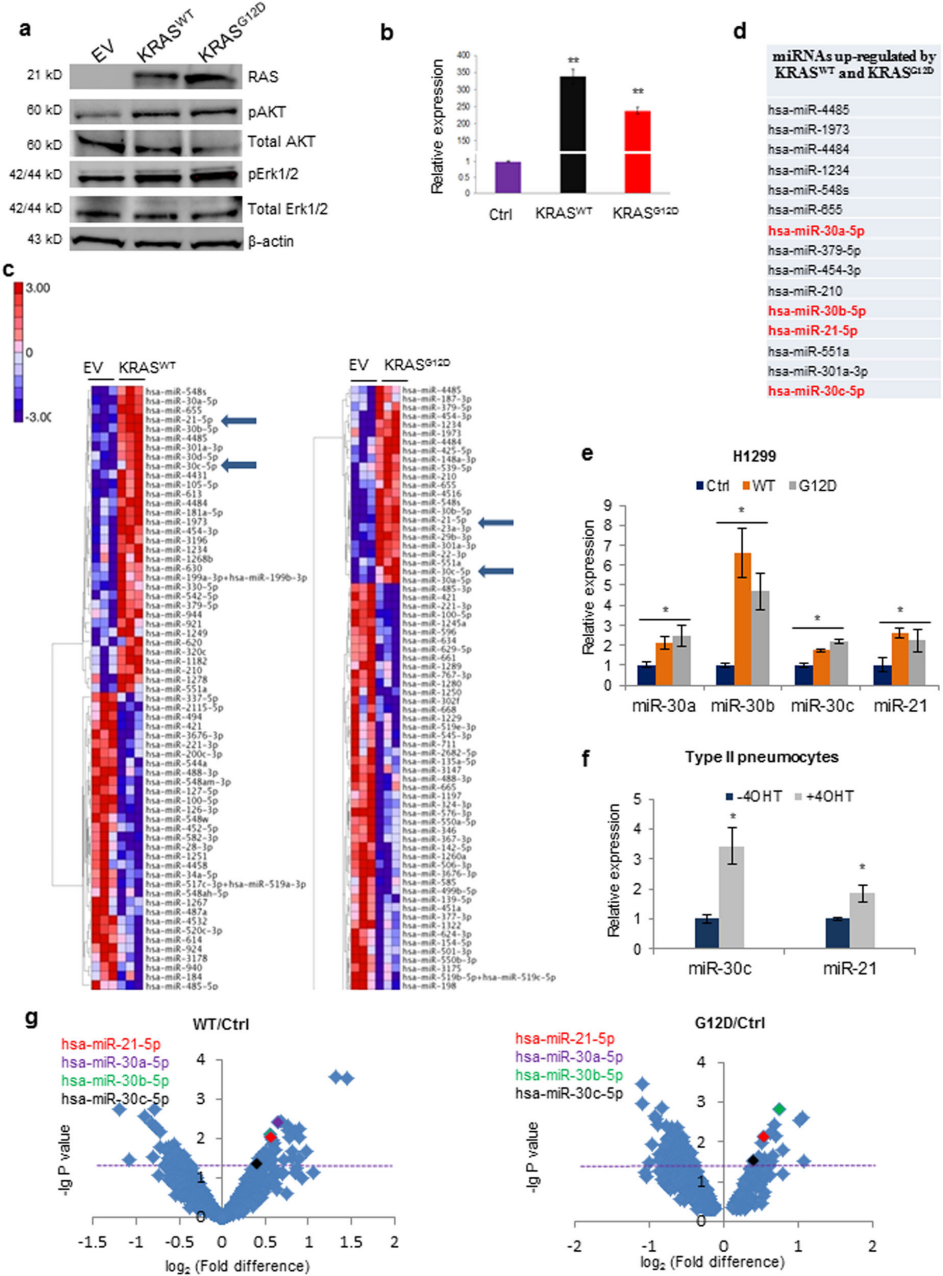
KRAS-modulated miRNAs. **a** Western blot showing AKT and ERKs activation after transient transfection of H1299 cells with KRAS^WT^ or KRAS^G12D^ **b** qPCR of KRAS^WT^ and KRAS^G12D^ in H1299 cells. **c** MiRNA heatmap of dysregulated miRNAs in H1299 cells overexpressing KRAS^WT^ or KRAS^G12D^. Complete heatmap for KRAS^G12D^ vs. control cells is reported in Supplementary Figure S1a. *P* values were obtained by ANOVA test (<0.05). **d** Table reporting common significantly upregulated miRNAs after KRAS^WT^ and KRAS^G12D^ overexpression. **e** MiR-30c and miR-21 expression in KRAS^WT^ and KRAS^G12D^ compared to control cells. **f** Increased levels of miR-30c and miR-21 in Type II pneumocytes after KRAS^G12V^ induction. **g** Volcano plots showing dysregulated miRNAs in KRAS^WT^ and KRAS^G12D^ vs. H1299 control cells. Highlighted with different colors are miR-30c family members and miR-21. Purple line indicates statistical significance. Bars indicate mean ± SD (*n* = 3) and the *P* values were addressed by two-tailed Student’s *t* test (*P < 0.05, **P < 0.001)

In order to identify KRAS-modulated miRNAs we first overexpressed KRAS wild type (KRAS^WT^) or KRAS^G12D^ in H1299 (KRAS^WT^) cells and overexpression was confirmed by immunoblotting and quantitative Real Time PCR (qPCR) (Fig. 1a,b). Interestingly, either KRAS^WT^ overexpression or mutation induced AKT and ERKs activation (Fig. 1a). Next, we examined the global miRNA expression profile using the NanoString technology. In total, 65 and 114 miRNAs were found differentially expressed (*P* < 0.05) in cells overexpressing KRAS^WT^ and KRAS^G12D^, respectively, compared to control cells (Fig. 1c,d; Supplementary Figure S1a). Real time PCR confirmed higher expression of the top modulated microRNAs in several KRAS^WT^ or KRAS^G12D^ transfected cell lines (Fig. 1e; Supplementary Figure S1b-d). Although we detected an increase in miR-210 and miR-655 after both KRAS^WT^ and KRAS^G12D^ overexpression (Supplementary Figure S1b), their basal expression levels were quite low. Consequently, we selected the top most upregulated microRNAs (miR-30 family and miR-21) for further characterization. Since members of miR-30 family share almost the same sequence and they potentially silence the same genes we chose to study miR-30c as representative member of the family. Upregulation of miR-30c and miR-21 was also detected in a normal immortalized KRAS^G12V^ inducible human cell line derived from alveolar epithelia^18,19^ and in a pancreatic KRAS inducible mouse cell line after treatment with doxycycline (Fig. 1f; Supplementary Figure S1e)^20^. Interestingly, these two microRNAs have been previously reported to be upregulated in pancreatic cancer^21^, a type of tumor with very high frequency of KRAS mutation^22^. Overexpression of KRAS^G12C^ also increased miR-30c and miR-21 in H1299 and A549 cells (Supplementary Figure S1f). H1299 cells harbor a mutation in NRAS, therefore to exclude a role for NRAS in miR-30c and miR-21 regulation, Calu-6 and A549 (NRAS wild type) cells were transfected with a mutant NRAS plasmid (NRAS Q61K). NRAS activation did not affect microRNA expression or ERKs activation in these cells (Supplementary Figures S1g, h). Furthermore, NRAS knockdown alone did not have any effect on miR-30c and miR-21 levels in H1299 cells whereas simultaneous silencing of NRAS and overexpression of KRAS^G12D^ did increase their levels, suggesting that the regulation of these microRNAs is exclusively KRAS dependent (Supplementary Figure S1i).

### MiR-30c and miR-21 target genes

**Fig. 2:**
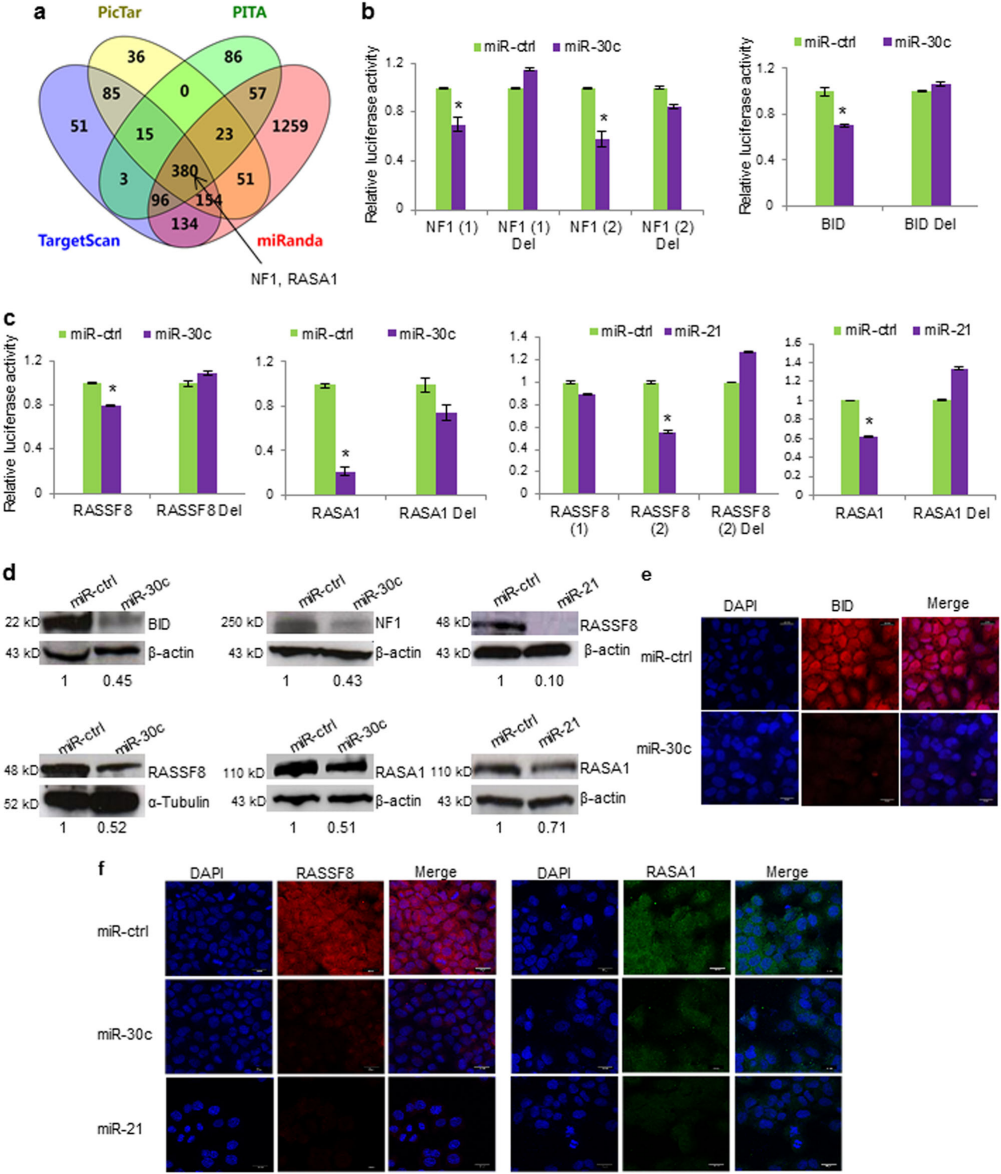
MiR-30c and miR-21 directly silence several tumor suppressor genes. **a** Bioinformatics prediction of matching seeded sequences in NF1, RASA1 3-UTRs using four different algorithms. **b**, **c** Luciferase assay in HEK293 cells cotransfected with miR-30c, miR-21 or a microRNA control (ctrl) and luciferase reporter constructs containing wild-type or mutated (Del) *NF1*, *BID*, *RASSF8* and *RASA1* 3′UTRs. Renilla luciferase activity was normalized to the firefly luciferase activity. **d** Enforced miR-30c and miR-21 expression decreased BID, NF1, RASSF8 and RASA1 endogenous levels in H1299 cells. Bands were quantified by densitometry using Image J software. **e**, **f** Immunofluorescence (IF) showing downregulation of miR-30c and miR-21 target genes in H1299 cells. Scale bar 20 μm. Bars indicate mean ± SD (*n* = 3) and the *P* values were addressed by two-tailed Student’s *t* test (*P < 0.05, **P < 0.001)

We used the online tool DIANA miRPath to identify miR-30c-modulated and miR-21-modulated pathways (Supplementary Figures S2a, b and Supplementary Table S1). MiR-30c and miR-21 were predicted to be involved in the regulation of oncogenic signaling downstream of KRAS such as PI3K/AKT and MAPK. Further, we conducted an in silico study using four different algorithms to predict miR-30c and miR-21 putative mRNA targets (Fig. 2a). Computational tools identified potential binding sites for miR-30c and miR-21 in the 3′UTRs of neurofibromin 1 (NF1), BH3-interacting domain death agonist (BID), Ras association domain-containing protein 8 (RASSF8), Ras p21 GTPase-activating protein 1 (RASA1) mRNAs (Supplementary Figure S3a). NF1 and RASA1 loss results in KRAS activation^23,24^. There is evidence that RASSF8 acts as a tumor suppressor gene in lung cancer by silencing NF-κB p65 (RelA) activation trough IKB-α^25^. BID, is a pro-apoptotic Bcl-2 protein which leads to activation of caspases^26^. All four search engines predicted potential binding sites for miR-30c in the 3′UTRs of NF1 and RASA1 mRNA (Fig. 2a; Supplementary Figure S3a). To verify a direct binding we carried out luciferase assays by cloning the 3′ UTRs of the target genes into a luciferase reporter vector. Co-transfection of these constructs along with miR-30c or miR-21 induced a significant reduction of the luciferase activity that was rescued when the miRNA-binding site was deleted by site direct mutagenesis (Fig. 2b,c and Supplementary Figure S3b). Overexpression of miR-30c and miR-21 led to a significant downregulation of BID, NF1, RASSF8, and RASA1 endogenous levels as assessed by western blot and immunofluorescence (Fig. 2d–f). However, enforced expression of miR-30c, and not of miR-21, decreased target mRNA levels (Supplementary Figures S4a-c) indicating that miR-30c regulates BID, NF1, RASA1, and RASSF8 at the transcriptional level while miR-21 halts RASA1 and RASSF8 protein expression.

### miR-30c and miR-21 regulate KRAS and NF-κB signaling

**Fig. 3:**
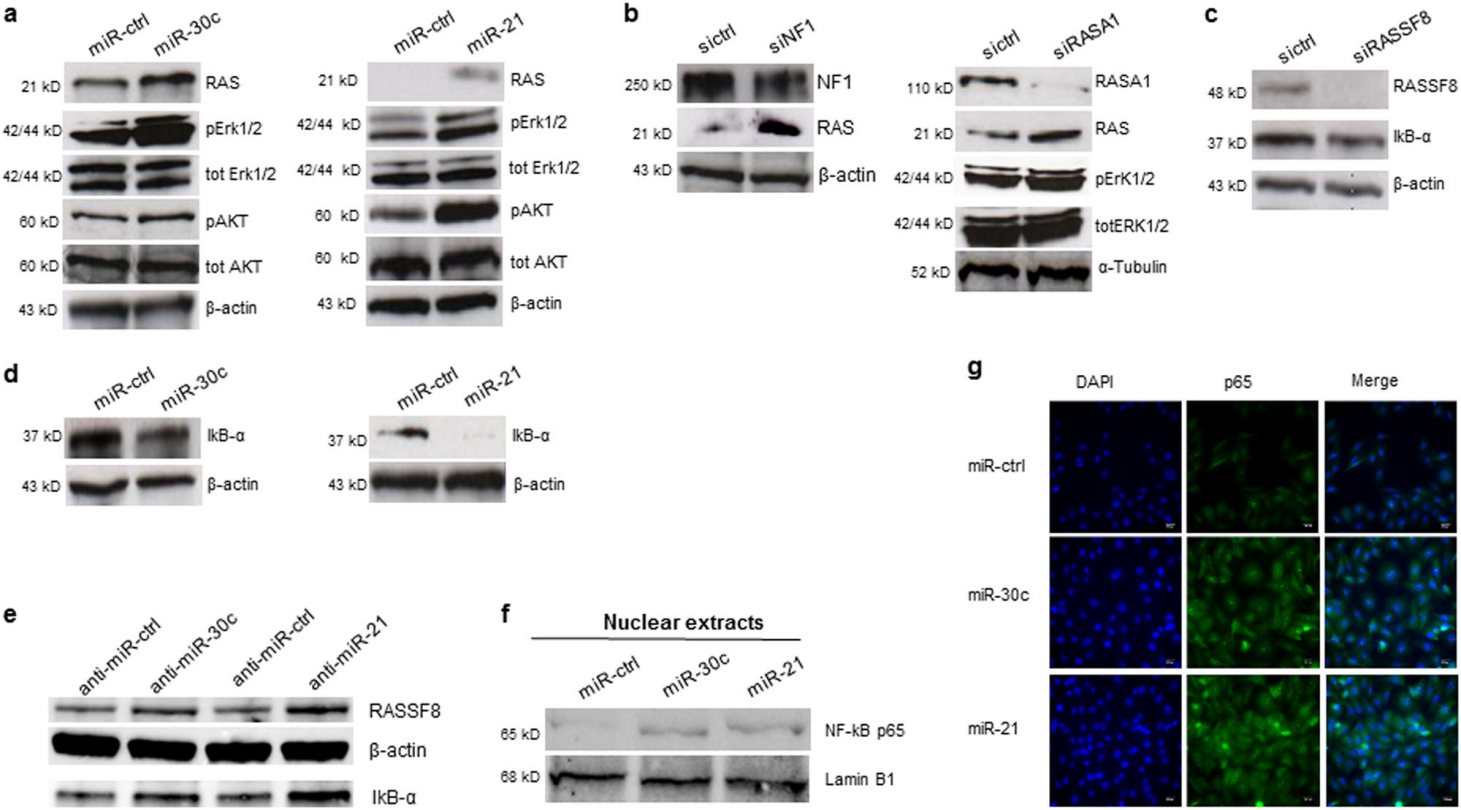
MiR-30c and miR-21 activate AKT, ERKs, and NF-κB signaling. **a** Activation of AKT and ERKs pathways after miR-30c and miR-21 enforced expression in H1299 cells. **b** NF1 and RASA1 silencing induced RAS upregulation and ERKs activation. **c** RASSF8 knockdown reduced IKB-α endogenous level. **d** MiR-30c and miR-21 activate NF-κB p65 by silencing IKB-α through RASSF8 downregulation. **e** MiR-30c and miR-21 knockdown increases RASSF8 and IKB-α endogenous level **f**–**g** NF-kBp65 nuclear localization after miR-30c and miR-21 enforced expression. Scale bar 200 μm

Since miR-30c and miR-21 silenced important KRAS regulators we tested whether these miRNAs had an effect on KRAS activation. Overexpression of miR-30c and miR-21 resulted in the upregulation of RAS endogenous levels and augmented ERK1/2 and AKT phosphorylation in H1299 cells (Fig. 3a). In line with these results, NF1 and RASA1 silencing gave rise to similar effects (Fig. 3b). It has recently been reported that RASSF8 is a NF-κB p65 negative regulator^25^. Therefore, we checked whether enforced expression of miR-30c and miR-21 could affect NF-κB signaling. In H1299 cells RASSF8-silencing reduced endogenous levels of IκB-α (Fig. 3c). Accordingly, same results were obtained after miR-30c and miR-21 enforced expression, as a consequence of RASSF8 knockdown (Fig. 3d) whereas anti-miR-30c and anti-miR-21 increased RASSF8 and IκB-α protein levels (Fig. 3e). Furthermore, forced increase of miR-30c and miR-21 resulted in the accumulation of NF-κB p65 in the nucleus, as assessed by immunofluorescence and immunoblot (Fig. 3f,g). In summary, these findings indicate that miR-30c and miR-21 not only activate KRAS signaling through the silencing of NF1 and RASA1 but also activate NF-κB signaling via RASSF8 downregulation.

### MiR-30c and miR-21 increase proliferation, invasion, and chemoresistance in NSCLC cell lines

**Fig. 4:**
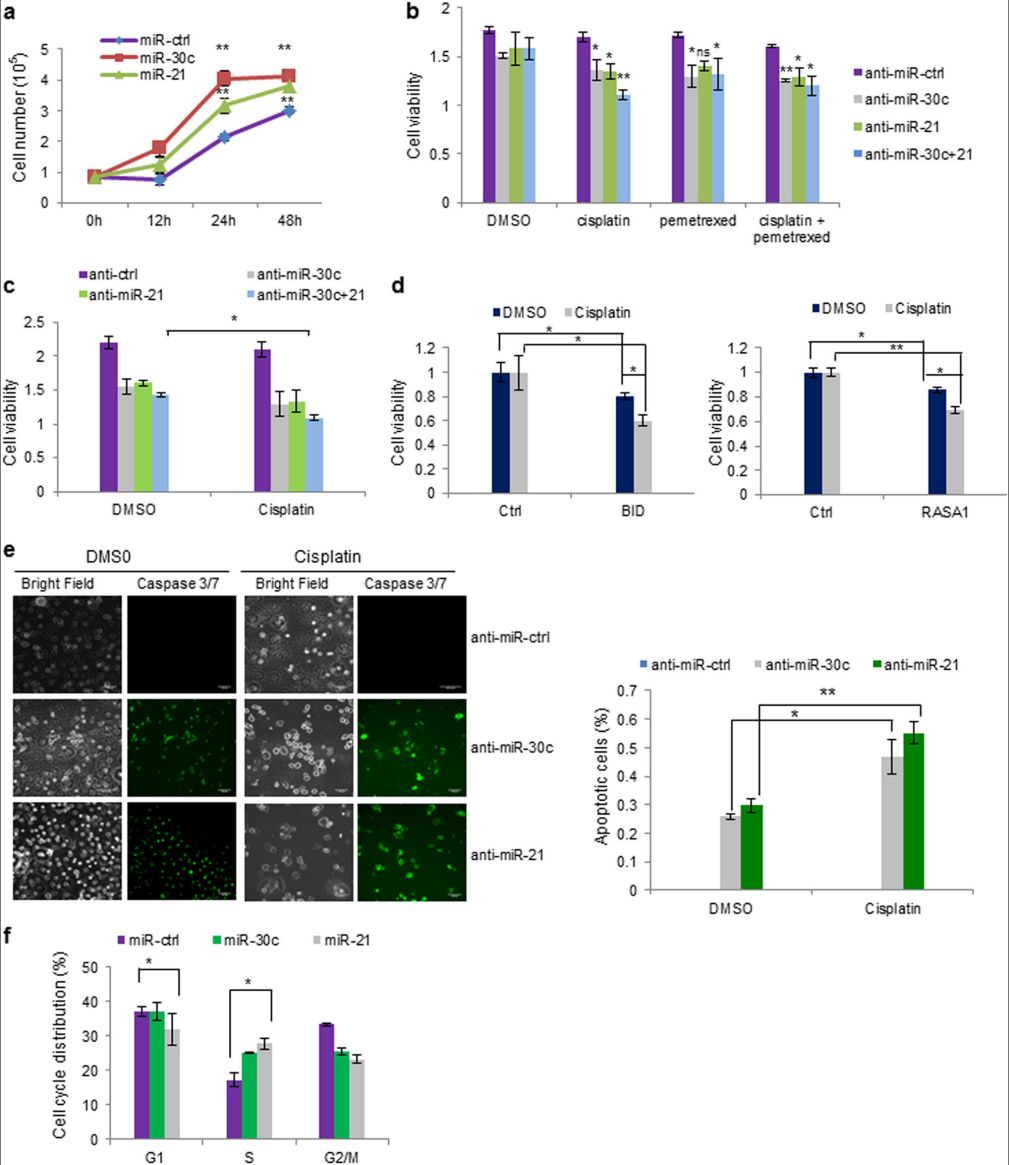
MiR-30c and miR-21 promote cell proliferation and increase drug resistance **a** Enforced expression of miR-30c and miR-21 promoted cell growth compared to control cells. **b** Cell viability assay after cisplatin, pemetrexed or combination cisplatin/pemetrexed, and miR-30c and/or miR-21 silencing in KRAS mutant A549 cells. **c** Effect of miR-30c and miR-21 silencing on cell proliferation in H1299 cells. **d** BID and RASA1 overexpression inhibits cell proliferation and increases sensitivity to cisplatin in A549 cells. **e** CellEvent caspase 3/7 assay showing increased apoptosis after miR-30c and miR-21 silencing and cisplatin treatment. Scale bar 200 µm. **f** Overexpression of miR-30c or miR-21 in H1299 cells induced an increase in the S phase of the cell cycle. Bars indicate mean ± SD (*n* = 3) and the *P* values were addressed by two-tailed Student’s *t* test (*P < 0.05, **P < 0.001)

It is known that KRAS plays an important role in cell proliferation, apoptosis, and drug resistance^2^ Ectopic expression of miR-30c and miR-21 significantly promoted cell growth of KRAS wild-type H1299 cells and reduced the response to cisplatin of H292 cisplatin-sensitive cells (Fig. 4a; Supplementary Figure S5a). Conversely, miR-30c or miR-21 silencing (Supplementary Figure S5b) inhibited cell proliferation in KRAS mutant A549 cells and reduced cisplatin resistance (Supplementary Figure S5c-d). We also examined the response to pemetrexed alone or as platinum doublet, frequently used as first line chemotherapy in patients with advanced NSCLC^27^ after miR-30c and/or miR-21 knockdown. MiR-30c and not miR-21 silencing increased the response to pemetrexed. Combination of anti-miR-30c and anti-miR-21 had a synergic effect in the response to cisplatin in both KRAS mutant and wild-type cells (Fig. 4b,c). In line with these findings, transient transfection of both BID and RASA1 sensitized A549 cells to cisplatin (Fig. 4d), suggesting that miR-30c and miR-21 exert their proliferative and oncogenic role by repressing these tumor suppressor genes. Furthermore, miR-30c and miR-21 knockdown induced apoptosis and this effect was significantly higher after cisplatin treatment (Fig. 4e). To determine whether an alteration in cell cycle progression was responsible for the promotion of cell proliferation by miR-30c and miR-21, we performed flow cytometry analysis. Transfection of miR-30c and miR-21 increased the number of H1299 cells in the S-phase compared to cells transfected with a negative control, denoting that miR-30c and miR-21 promote DNA synthesis in NSCLC cells (Fig. 4f). Accordingly, cell cycle analysis after KRAS-silencing induced a G1 arrest (Supplementary Figures S6a, b). In summary, miR-30c and miR-21 induce cisplatin resistance by silencing BID and RASA1 and increase the proliferation rate of NSCLC cells by regulating cell cycle progression. Next, we determined the effect of miR-30c and miR-21 overexpression or corresponding target genes silencing on migratory and invasive capabilities of NSCLC cells. MiR-30c and miR-21 enforced expression promoted migration and invasion in H1299 cells (Supplementary Figure S7a). Inhibition of RASA1 fostered both migration and invasion (Supplementary Figure S7b, c), whereas downregulation of NF1 and RASSF8 increased migration and had a minor but significant effect on invasion (Supplementary Figure S7d). Downregulation of SNAIL and several other mesenchymal markers was identified after miR-30c and miR-21 knockdown (Supplementary Figure S7e, f), therefore miR-21 and miR-30c are involved in the control of the epithelial–mesenchymal transition (EMT) in lung cancer. Importantly, silencing of RASA1, NF1, and RASSF8 increased the expression of two mesenchymal markers, AP4 and SNAIL, confirming that miR-30c and miR-21 regulate EMT through these target genes (Supplementary Figures S7g, h).

### ELK1 transcriptionally activates miR-30c and miR-21

**Fig. 5:**
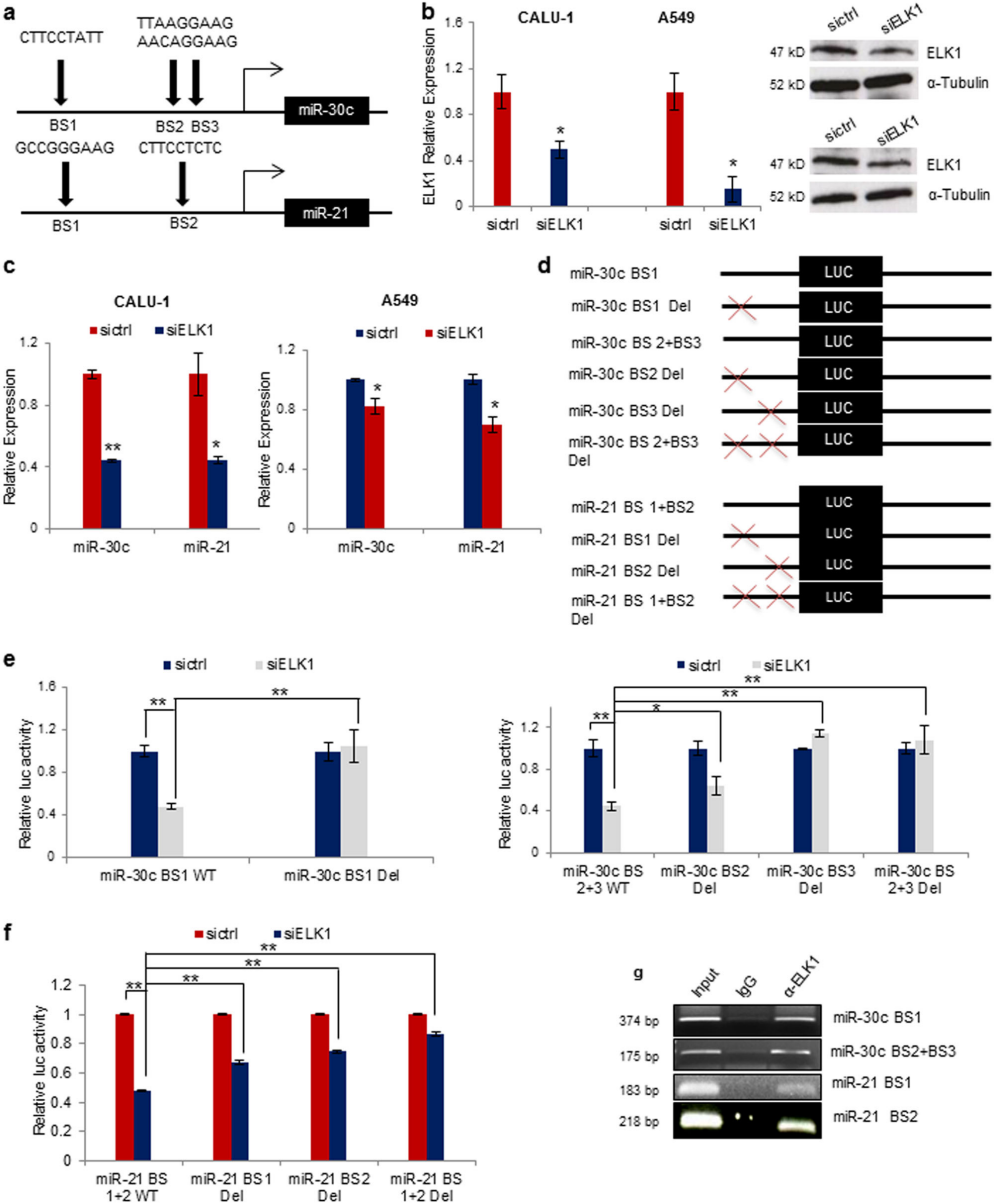
ELK1 directly binds to miR-30c and miR-21 proximal promoter regions. **a** Schematic representation of ELK1-binding sites in miR-30c and miR-21 promoters. **b**, **c** ELK1 knockdown reduced miR-30c and miR-21 expression in A549 and Calu-1 cells. (Fig. 5b top panel, Calu-1 cells; bottom panel, A549 cells) **d** Schematic representation of ELK1-binding site deletions. **e**, **f** ELK1 silencing reduced luciferase activity whereas deletion of the binding sites rescued luciferase activity. **g** ChIP showing a direct interaction between ELK1 and miR-30c and miR-21 promoter regions. Bars indicate mean ± SD (*n* = 3) and the *P* values were addressed by two-tailed Student’s *t* test (*P < 0.05, **P < 0.001)

Next, we investigated the mechanism through which KRAS could promote miR-30c and miR-21 activation. Raf/MEK/ERK pathway stimulates the ETS-domain transcription factor ELK1 which regulates the transcription of immediate early response genes^28^. Using PROMO 8.3 algorithm we found three different putative ELK1-binding sites in miR-30c and two ELK1-binding sites in miR-21 promoter (Fig. 5a)^29^. ELK1 silencing (Fig. 5b) or treatment with the MEK inhibitor Trametinib decreased miR-30c and miR-21 expression levels in two different NSCLC cell lines (Fig. 5c; Supplementary Figure 7i). To verify whether ELK1 was the transcription factor involved in miR-21 and miR-30c activation, two miR-30c promoter regions containing one and two ELK1-binding sites, respectively, and one region containing two ELK1-binding sites spanning miR-21 promoter, were cloned in a promoterless reporter vector (Fig. 5d). ELK1 knockdown reduced miR-30c and miR-21 promoter activity (Fig. 5e,f) whereas deletion of the binding sites by site direct mutagenesis rescued this effect (Fig. 5d–f). Chromatin immunoprecipitation (ChIP) assay showed that endogenous ELK1 was enriched at the binding sites of both promoters (Fig. 5g). These results indicate that KRAS transactivates miR-30c and miR-21 through the downstream activation and recruitment of ELK1 to the miRNAs’ proximal promoters.

### MiR-30c and miR-21 expression and modulation in vivo

**Fig. 6:**
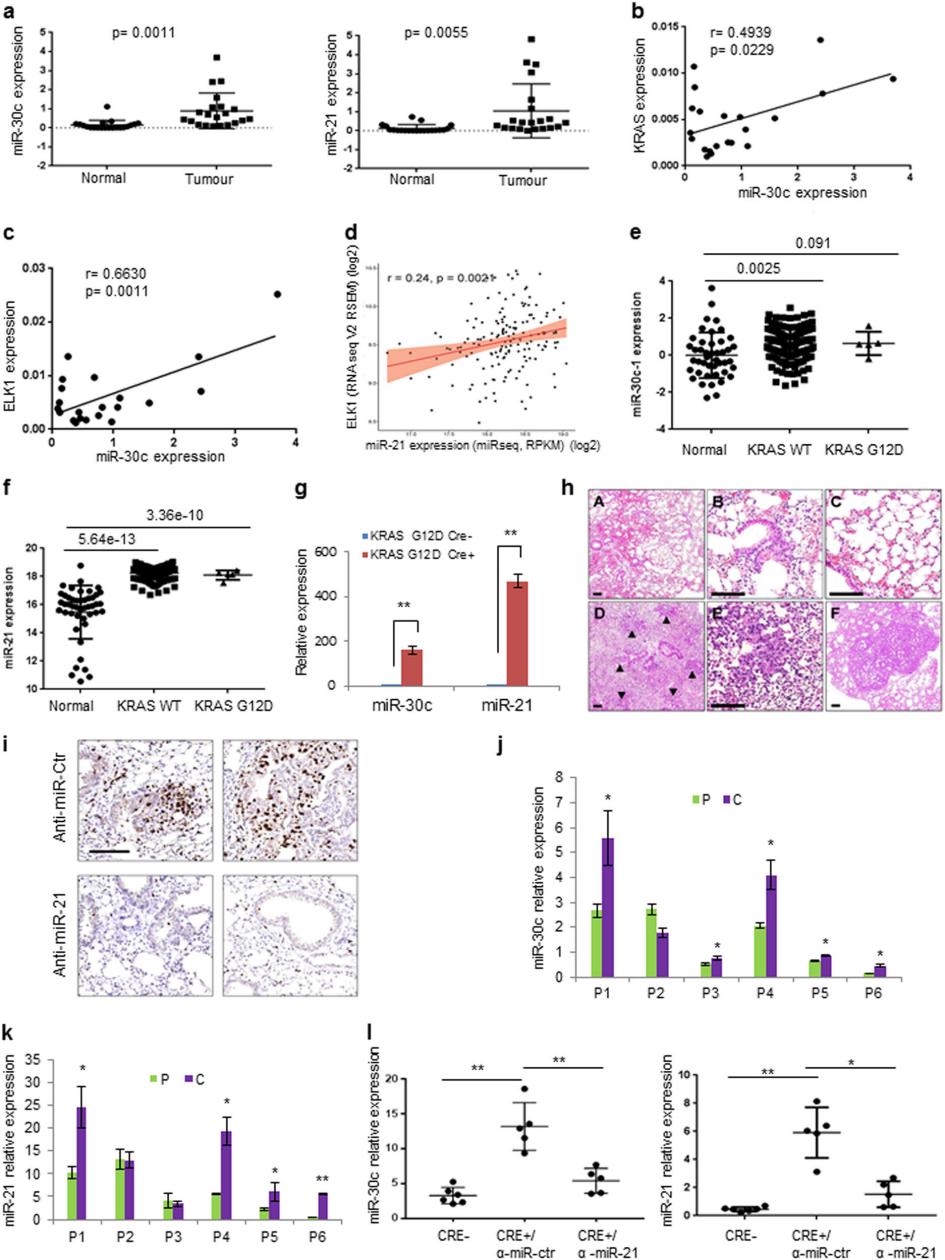
MiR-30c and miR-21 promote tumorigenesis in vivo. **a** MiR-30c and miR-21 upregulation in tumor samples compared to normal lung (tumor samples *n* = 21; normal lung *n* = 21). **b**, **c** KRAS and ELK1 directly correlate with miR-30c in lung tumors (tumor samples *n* = 21). **d** Pearson correlation between Elk1 and miR-21 in 160 (155 KRAS^WT^ and 5 KRAS^G12D^) adenocarcinoma samples. **e**, **f** MiR-30c (44 normal samples, 150 lung adenocarcinoma KRAS^WT^, and 5 lung adenocarcinoma KRAS^G12D^) and miR-21 (46 normal samples, 155 lung adenocarcinoma KRAS^WT^, and 5 lung adenocarcinoma KRAS^G12D^) expression in tumors expressing KRAS^WT^ and KRAS^G12D^ compared to normal samples from the TCGA data set LUAD. **g** MiR-30c and miR-21 are significantly upregulated in tumors from KRAS^G12D^ mice compared to control mice. **h** Representative pictures of the effect of LNA-anti-miR-21 (**a**–**c**) and LNA-anti-miRNA control (**d**–**f**) treatment 12 weeks post adenoCRE inhalation in KRAS^G12D^ transgenic mice. Lungs from mice treated with anti-miR-21 presented a normal alveolar texture with normal bronchiolar structures and no or minimal signs of fibrosis/flogosis (**a**). Normal representation of bronchiolar epithelial cells (**b**) and of alveolar framework (**c**) is shown. KRAS^G12D^ mice treated with anti-miR control developed the usual preneoplastic lesions observed in this model. A significant disarrangement of the pulmonary parenchyma (**d**), with the accumulation of preneoplastic lesions such as atypical adenomatous hyperplasias (**e**) or adenomas (**f**; the largest adenoma is showed). Scale bars 100 µm. **i** KI67 in lungs from mice treated with anti-miR-ctr and anti-miR-21. **j**, **k** qPCR showing miR-30c and miR-21 levels in plasma samples obtained from patients undergoing surgical resection of NSCLC. P peripheral vein, C cancer draining pulmonary vein, P1–P6 patient 1–6. **l** MiR-30c and miR-21 expression levels in plasma from KRAS^G12D^ mice treated with anti-miR-ctr or anti-miR-21. Bars indicate mean ± SD (*n* = 3) and the *P* values were addressed by two-tailed Student’s *t* test (*P < 0.05, **P < 0.001)

Kaplan–Meier analysis based on the average expression of miR-30c and miR-21 target genes was performed to predict the risk for NSCLC patients using Kmplot database^30^. High expression of BID, NF1, RASSF8, and RASA1 was associated with longer survival whereas high KRAS and ELK1 level correlated with a shorter lifespan (Supplementary Figures S8a,b). Further, we checked the expression of miR-30c and miR-21 in 21 adenocarcinomas and corresponding normal counterpart. MiR-30c and miR-21, as well KRAS and ELK1 expression, was significantly elevated in tumors compared to normal lung samples (Fig. 6a and Supplementary Figure S9a). A significant positive correlation between miR-30c and KRAS or ELK1 was observed in the 21 lung tumor samples (*P* = 0.0229 and *P* = 0.0011, respectively) (Fig. 6b,c) and between miR-21 and ELK1 in 160 adenocarcinoma samples from the TCGA (LUAD data set) (Fig. 6d). Furthermore, miR-30c and miR-21 were found upregulated in KRAS^WT^ (miR-30c, sample *n* = 150; miR-21, sample *n* = 155) and KRAS^G12D^ mutant lung adenocarcinoma (miR-30c, sample *n* = 5; miR-21, sample *n* = 5) compared to normal lung (miR-30c, sample *n* = 44; miR-21 sample *n* = 46) (Fig. 6e,f). Overexpression of KRAS and ELK1 was confirmed in these tumors compared to normal lung samples (Supplementary Figures S9b, c). Subsequently, we analyzed the expression of miR-30c and miR-21 in vivo in a mouse model of lung cancer (Kras^LSL-G12D^) that involves activation of an oncogenic *Kras* allele (*Kras*^*G12D*^) following intranasal administration of plaque-forming units of a recombinant adenovirus-expressing Cre recombinase (AdenoCre)^31^. These mice develop lung adenomas with 100% penetrance that eventually progress to high-grade adenocarcinomas^32^. Seven weeks after infection with AdenoCre, mice were killed and expression of miR-30c and miR-21 was analyzed in the lungs of these mice and in the lungs of mice that were infected with GFP adenovirus as control. Both miRNAs were substantially increased in the lungs of mice treated with AdenoCre, suggesting that KRAS may exert its tumorigenic function through these two miRNAs at very early stages of lung cancer (Fig. 6g). Since we have previously shown the therapeutic potential of miR-30c^33^, we tested whether modulation of miR-21 could have an effect on lung tumorigenesis in vivo. Kras^LSL-G12D^ mice received systemically LNA-anti-miR-21 once per week for 7 weeks and two doses of cisplatin intraperitoneally for all the length of the experiment. In vivo silencing of miR-21 completely abolished the appearance of adenomas and hyperplasia compared to mice treated with anti-miR-control (Fig. 6h,i; Supplementary Figure S9d). Finally, we analyzed miR-30c and miR-21 expression in a unique set of plasma samples obtained from patients undergoing surgical resection of early stage NSCLC. miR-30c and miR-21 expression was analyzed in plasma from a peripheral vein (P) (taken before the operation) and in plasma from the pulmonary vein directly draining a cancer-bearing lobe (C) (taken during surgery) (Supplementary Figure 9Se). As shown in Fig. 6j,k, miR-30c and miR-21 expression levels were significantly higher in C compared to P and in the matched tumor/normal tissue of the same patients (Fig. 6j,k; Supplementary Figures S9f, g). MiR-30c and miR-21 levels were also upregulated in the blood from KRAS^LSL-G12D^ mice treated with anti-miR-ctrl compared to mice treated with anti-miR-21 (Fig. 6l). Taken together, our findings suggest that miR-30c and miR-21 are upregulated in NSCLC in the early stage of NSCLC development and released into the bloodstream. Therefore, the combination miR-21/30c could be exploited as potential biomarker for early detection of lung cancer.

## Discussion

**Fig. 7:**
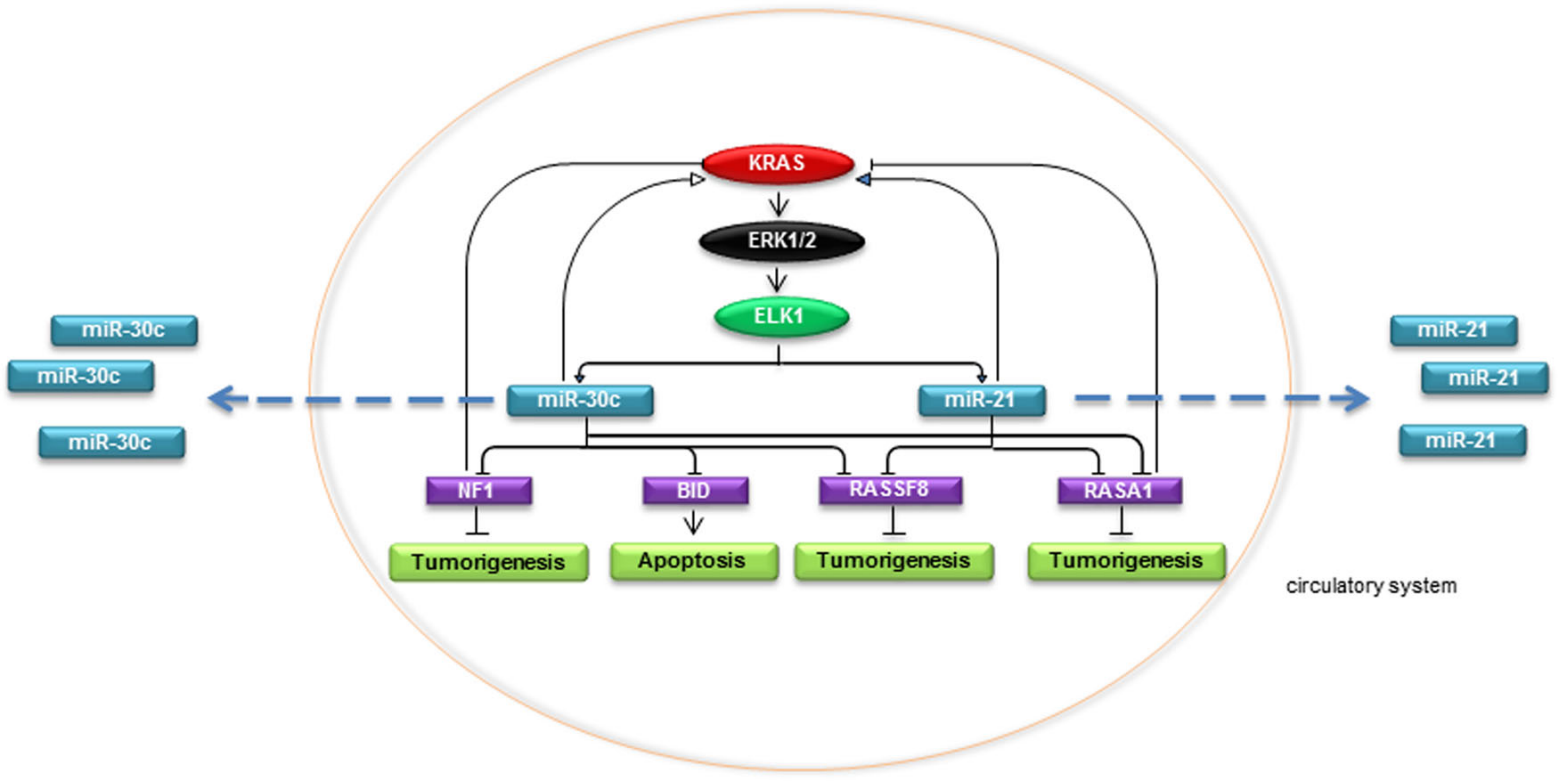
Working model. KRAS, through the transcription factor ELK1, activates miR-30c and miR-21, which in turn, by downregulating NF1, RASA1, RASSF8, and BID, regulates KRAS, NF-kB, and the intrinsic apoptotic pathways, inducing lung tumorigenesis and inhibiting apoptosis in NSCLC. MiR-30c and miR-21 are released into the bloodstream and could be potential biomarkers for early NSCLC detection

Despite decades of study, direct KRAS targeting still remains unsuccessful in the clinical setting, thus blocking KRAS effector pathways is an attractive therapeutic option. Because RAS activates PI3K/AKT and ERKs/mTOR signaling, many combination therapies have been tested^34^. However, these combinations have led to a modest improvement in treatment effect and have shown to be highly toxic in patients^35^. To date several reports demonstrated that miRNAs work as oncogenes or tumor suppressor genes and their modulation can induce drug resistance or sensitivity^36,37^. MiRNAs are attractive as therapeutic tools because they can silence multiple gene(s) and therefore switch off simultaneously different pathways, which is not possible with protein-based drugs, with eventually lower toxicity compared to drug combinations. Hitherto, a direct comparison of microRNA expression profiling of KRAS wild type and KRAS mutant forms in NSCLC has not been performed. In this study, we investigated the role of KRAS-regulated miRNAs and focused on microRNAs that were commonly modulated by KRAS^WT^ and KRAS^G12D^. In agreement with Horsch and collaborators, who previously showed that both overexpression and mutations of KRAS led to common altered gene expression patterns, we identified microRNAs commonly modulated by both KRAS^WT^ and KRAS^G12D^^38^. In fact, either KRAS^WT^ overexpression or mutation induced AKT and ERKs activation (Fig. 1a). We focused on the top modulated and highly expressed microRNAs and found that miR-30c directly silenced NF1, BID, RASSF8, and RASA1, whereas miR-21 inhibited RASSF8 and RASA1. Ras-GTPase-activating proteins (RasGAPs) are negative regulators of RAS and ERK/MAPK pathway. Very recently it has been reported that loss of function mutations in RASA1 and NF1 is a strong mitogenic driver in NSCLC, suggesting that loss of more than one RasGAP is a major determinant in lung tumor survival^39^. Furthermore, low expression of NF1 is a marker of erlotinib resistance^40^. We show that KRAS silences NF1/RASA1 expression in lung tumors via microRNAs modulation, establishing a negative feedback loop that leads to increased tumorigenesis and drug resistance. miR-30c and miR-21 fostered proliferation and reduced response to cisplatin by targeting BID and RASA1^41,42^ while promoted invasive capabilities and EMT of NSCLC cells through RASA1, NF1, and RASSF8 downregulation. RASGAPs should exert a tumor suppressor function in KRAS wild type and not in cells that harbor a mutant KRAS allele. We found that RASA1 enforced expression reduced cell proliferation and increased the response to cisplatin in KRAS mutant lung cancer cells. This could be due to the suppressive effect that RASA1 exerts on KRAS downstream signalings, such as the Rho pathway, as previously described^43,44^. Our experiments also pointed out that miR-30c and miR-21 are exclusively modulated by KRAS and not NRAS as they might perform distinct functions during transformation^45^. Forced expression of mutant NRAS in different cell lines revealed that NRAS does not increase ERKs phosphorylation, fundamental for ELK1 and accordingly miR-30c and miR-21 activation. We previously reported that miR-30c and miR-21 are regulated by the epidermal growth factor receptor and induce resistance to tyrosine kinase inhibitors^33^. This work defines that miR-30c and miR-21 are specifically activated by KRAS and play an important role in lung cancer development and chemoresistance by targeting crucial tumor suppressor genes (Fig. 7). Importantly, miR-30c and miR-21 were upregulated in a large cohort of NSCLC samples compared to the normal counterparts and in the lungs from a KRAS mouse model, therefore these two miRNAs are KRAS-modulated oncogenes also in vivo. Systemic delivery of LNA-anti-miR-21 combined with only two doses of cisplatin over 7 weeks in these mice completely abolished the onset of lung adenomas and hyperplasia, denoting that miR-21 plays a major role in KRAS-mediated lung tumorigenesis. Early cancer detection remains the best option for cancer treatment. MicroRNAs are released in the bloodstream, where they are considerably stable, embedded in exosomes, microvesicles or apoptotic bodies and in high-density and low-density lipoproteins^46–48^. Several studies have shown the potential of microRNAs as non-invasive prognostic and diagnostic biomarkers to predict disease onset or progression^49–51^. However, these studies lack endogenous controls from the same patient, which would drastically improve their reliability. Using a unique set of plasma samples from patients undergoing surgical resection of early NSCLC and a mouse model of lung cancer we verified that miR-30c and 21 are upregulated in tumors compared to the normal counterpart and then released into the circulation (Fig. 7). In conclusion, this study sheds light on the molecular mechanisms through which wild type or mutated KRAS induces tumorigenesis. From a therapeutic perspective, our findings indicate that miR-30c and miR-21 inhibition halts lung tumorigenesis in vitro and in vivo by switching off simultaneously KRAS effector signalings such as PI3K/AKT, ERKs, and the NF-κB pathways and that these microRNAs could be potential biomarkers for NSCLC early detection and to stratify KRAS-driven NSCLC.

## Material and methods

### Cell lines and reagents

All cell lines used in this study were purchased from ATCC or identification was performed on established lines using PowerPlex® 21 System (Promega). Lines were tested for Mycoplasma every 3 months using the Venor®*GeM* Classic Mycoplasma PCR Detection Kit (Cambio Ltd). H1299, A549, Calu-6, H1703, H292, and Type II pneumocyte cells were maintained in RPMI 1640 medium, whereas Calu-1, HEK293, and iKRAS cells were grown in DMEM medium. An aliquot of 500 μM 4-OHT (48 h) and 100 ng/ml of doxycycline (48 h) were used to induce KRAS in Type II pneumocytes and iKRAS cells, respectively. MicroRNA mimics, anti-miRNAs, and siRNAs were purchased from Ambion. BID, RASA1, and NRAS Q61K plasmids were obtained from Addgene, KRAS wild type and KRAS^G12D^ were purchased from Origene.

### Nanostring

The Nanostring nCounter Human v2 miR Expression Assay kit (NanoString, Seattle, Washington, USA) was used to profile more than 800 human and human-associated viral miRNAs as previously described^13^.

### Luciferase reporter assay

3′UTRs of NF1, RASA1, BID, and RASSF8 containing miR-30c- or miR-21-binding sites were PCR amplified and inserted into the pGL3 control vector (Promega). Deletions of miRNA-binding sites were performed using a Quick-Change Mutagenesis Kit (Stratagene). HEK293 cells were cotransfected with 200 ng of pGL3–3′UTRs plasmids, 20 ng of Renilla plasmid (Promega), and 50 nM of microRNA using Lipofectamine 2000 (Invitrogen). Cells were collected 24 h post-transfection and assayed with Dual Luciferase Assay (Promega) according to the manufacturer’s instructions. Promoter regions of miR-30c and miR-21 containing ELK1 putative-binding sites were PCR amplified and inserted into the promoterless pGL3 basic vector (Promega). A549 cells were transfected with Lipofectamine 2000 (Invitrogen), 200 ng of pGL3 basic empty vector or 200 ng of pGL3 basic containing the above genomic fragments, 20 ng of Renilla (Promega) and 50 nM of ELK1 siRNA. Forty-eight hours after transfection cells were lysed and assayed with Dual Luciferase Assay (Promega) according to the manufacturer’s instructions. Primers used to amplify 3′ UTRs of miR-30c and miR-21 target genes or delete the microRNA-binding sites and primers used to amplify miR-30c and miR-21 promoter regions are reported in Supplementary Tables S2 and S3, respectively.

### Immunofluorescence

Cells cultured on coverslips were transfected with miR-30c, miR-21 or control miR for 48 h and then fixed with 4% paraformaldehyde for 15 min, permeabilized in 0.2% Triton X-100 /PBS for 20 min, and incubated in primary antibodies for 1 h. Cells were stained with DAPI (ThermoFisher Scientific) and imaged using confocal microscope (Leica).

### Cell viability assay

In total, 5 × 10^3^ cells were cultured in 96-well plate and transfected with miRNA mimics or siRNAs and treated with cisplatin (10 μM, Sigma) and/or pemetrexed, Selleck Chemicals). Cell viability was determined using the Cell Titer 96 Aqueous One solution Cell viability assay (Promega) according to the manufacturer’s instructions. Metabolically active cells were detected by adding 20 μl of MTS reagent to each well and incubated at 37 °C for 1 h. Absorbance at 490 nm was analyzed in a Multilabel Counter (SpectraMax M5).

### Cellevent caspase 3/7 assay

Cells were grown on coverslips in six-well plates and transfected with anti-miR-ctrl, anti-miR-30c, or anti-miR-21. Cisplatin (10 μM) was added to the media 48 h after transfection. 72 h after transfection cells were incubated with 2 µM of CellEvent Caspase 3/7 Green Detection Reagent (ThermoFisher Scientific) for 30 min. Coverslips were then washed twice with PBS and ProLong Diamond antifade reagent was used to fix the cells overnight. Images were taken by confocal microscope (Leica) at ×20 magnification.

### Migration and invasion assay

H1299 cells were transfected with miR-30c, miR-21, or NF1, RASSF8 and RASA1 siRNAs for 48 h using Lipofectamine 2000 (Invitrogen). Transwell insert chambers with 8-μm porous membrane (Corning) were used for the assay. Cells were added to the top chamber in serum free media and migration/invasion assays were performed as described in ref. ^52^.

### Cell growth assay

A total of 8.4 × 10^4^ cells were seeded in 12-well plate and transfected with miRNA control or miR-30c and miR-21 for 12, 24 or 48 h. Cells were counted using Count II FL (Life Technologies) at different time points.

### Cell cycle analysis

Cells were transfected using Lipofectamine 2000, washed with PBS, fixed with ice-cold 70% ethanol, and incubated overnight at −20 °C. For cell cycle analysis, cells were stained with propidium iodide (PI) staining buffer (PBS, 0.1% Triton X-100, 0.5 mg/ml PI) and incubated at 37 °C for 1 h. Results were obtained using 4 color Calibur device (BD) and analyzed with Flowjo software (Flowjo Enterprise).

### In vivo studies

All procedures involving animals were approved by CRUK Manchester Institute’s Animal Welfare and Ethical Review body in accordance with the Animals Scientific Procedures Act 1986 and according to the ARRIVE guidelines and the Committee of the National Cancer Research Institute guidelines and conducted under project license number P72E31537 (M.G.). KRAS^LSL-G12D^ mice were divided in three groups (five mice per group). Group 2 and 3 only received adenoCRE recombinase by intranasal inhalation at 6 weeks of age. Five weeks after adenoCRE administration mice in group 2 were treated with anti-miR-ctrl and mice in group 3 with anti-miR-21 once per week for 7 weeks plus two doses of cisplatin intraperitoneally (i.p.). Twelve weeks after initial adenoCRE inhalation mice were euthanized, lungs were weighted, tumors, and normal lung were harvested and blood collected for analysis.

### Plasma and normal/tumor lung samples

Blood was obtained from patients undergoing definitive surgical resection of NSCLC at the Department of Thoracic Surgery, University Hospital of South Manchester, UK. All patients provided written informed consent prior to study participation. Patients were excluded from the study if they had any previous diagnosis of malignancy within 5 years or had received any cancer treatment prior to surgery. All patients had blood taken from a peripheral vein before surgery. During the operation, but prior to any tumor sampling/vessel ligation or lung resection, blood was taken from the pulmonary vein draining the cancer-bearing lobe. Samples of resected tumor and normal lung tissue were collected from participants. Blood was collected and processed using standard operating procedures within 30 min of blood draw. Resected lung specimens were sampled from tumor and macroscopically normal tissue by a specialist Thoracic Pathologist and stored at −80 °C within 1 h of resection.

### MicroRNA extraction from plasma samples

MiRNAs were extracted from 200 μl plasma using the miRNeasy kit (Qiagen) according to the manufacturer’s instructions. A synthetic plant (Arabidopsis thaliana, Life Technologies) miR-159 “spike-in” (12.5 pM) was added as an exogenous control.

### Chromatin immunoprecipitation

Cells were cultured to optimal confluence and cross-linked with formaldehyde 10 min at 37 °C and washed with ice-cold 1 × PBS, scraped in 1 × PBS plus protease inhibitors, and collected by centrifugation. Cell pellets were then sonicated. DNA-protein complexes were immunoprecipitated using 2 μg of ELK1 antibody. The purified chromatin was subjected to PCR and the products were analyzed by gel electrophoresis using a 2% agarose gel. Primers used as reported in Supplementary Table 4.

### RNA extraction and real time quantitative PCR

Total RNA was extracted using TRIzol solution, according to the manufacturer’s Instructions. qPCR was performed as described in ref.^37^. Primers are listed in Supplementary Table S5.

### Western blotting

Western blot was performed as described in ref.^31^.Antibodies are listed in the Supplementary Table S6.

### Human tumor and normal lung specimens

Primary NSCLC and normal lung samples were provided by the Department of Pathology, The Ohio State University (OSU). All human tissues were obtained according to a protocol approved by the Ohio State Institutional Review Board. Tissue samples were fresh-frozen in liquid nitrogen after surgery and kept at −80 °C. Frozen tissue samples were homogenized using the Tissue Ruptor (Qiagen) before RNA extraction. Total RNA was extracted using Trizol (Invitrogen) in accordance with the manufacturer’s instructions.

### In silico analysis

In silico prediction of miRNA targets was performed with TargetScan^53^, PicTar^54^, miRanda^55^, and PITA^56^ algorithms using the HUGO gene symbol as common identifier. Level 3 TCGA datasets of miRNA expression in 532 lung adenocarcinoma (LUAD) patients were downloaded from The Broad Institute Genome Data Analysis Center (GDAC) [doi:10.7908/C11G0KM9]. Analysis was performed on the available samples expressing miR-30c (normal lung samples *n* = 44, lung adenocarcinoma KRAS WT samples *n* = 150 and lung adenocarcinoma KRAS G12D samples *n* = 5) or miR-21 (normal lung samples *n* = 46, lung adenocarcinoma KRAS WT samples *n* = 155 and lung adenocarcinoma KRAS G12D samples *n* = 5) using GraphPad Prism package (GraphPad Software Inc). Kaplan–Meier Plotter software was used to analyze and generate survival time data relative to high or low expression of miR-30c and miR-21 target genes^30^. Survival plots were generated from http://kmplot.com/analysis/. DIANA miRPath v3.0 software was employed to analyze miR-30c and miR-21-related signaling pathways and number of target genes based on TarBase v7.0 data set^57^.

### Statistical analysis

Significance was determined by two-tailed Student’s *t* test. *P < 0.05 and **P < 0.001 was defined as statistically significant. The Pearson’s correlation was calculated using the GraphPad Prism package (GraphPad Software Inc).

## Electronic supplementary material

Supplementary Information
