# Supplementary material for: KRAS induces lung tumorigenesis through microRNAs modulation
Source: Cell Death Dis. 2018 Feb 13;9(2):219. doi: 10.1038/s41419-017-0243-9 (PMC5833396; doi:10.1038/s41419-017-0243-9)
Supplement: Supplementary file 1 — Supplementary Information [file 41419_2017_243_MOESM1_ESM.pdf]

Supplementary Information

K-RAS induces lung tumorigenesis through microRNAs modulation.

Lei Shi, Justin Middleton, Young-Jun Jeon, Peter Magee, Dario Veneziano, Alessandro Lagana', Hui-Sun Leong, Sudhakar Sahoo, Matteo Fassan, Richard Booton, Rajesh Shah, Philip A. J. Crosbie and Michela Garofalo.

Supplementary Figures S1-S9
Supplementary Tables S1-S6

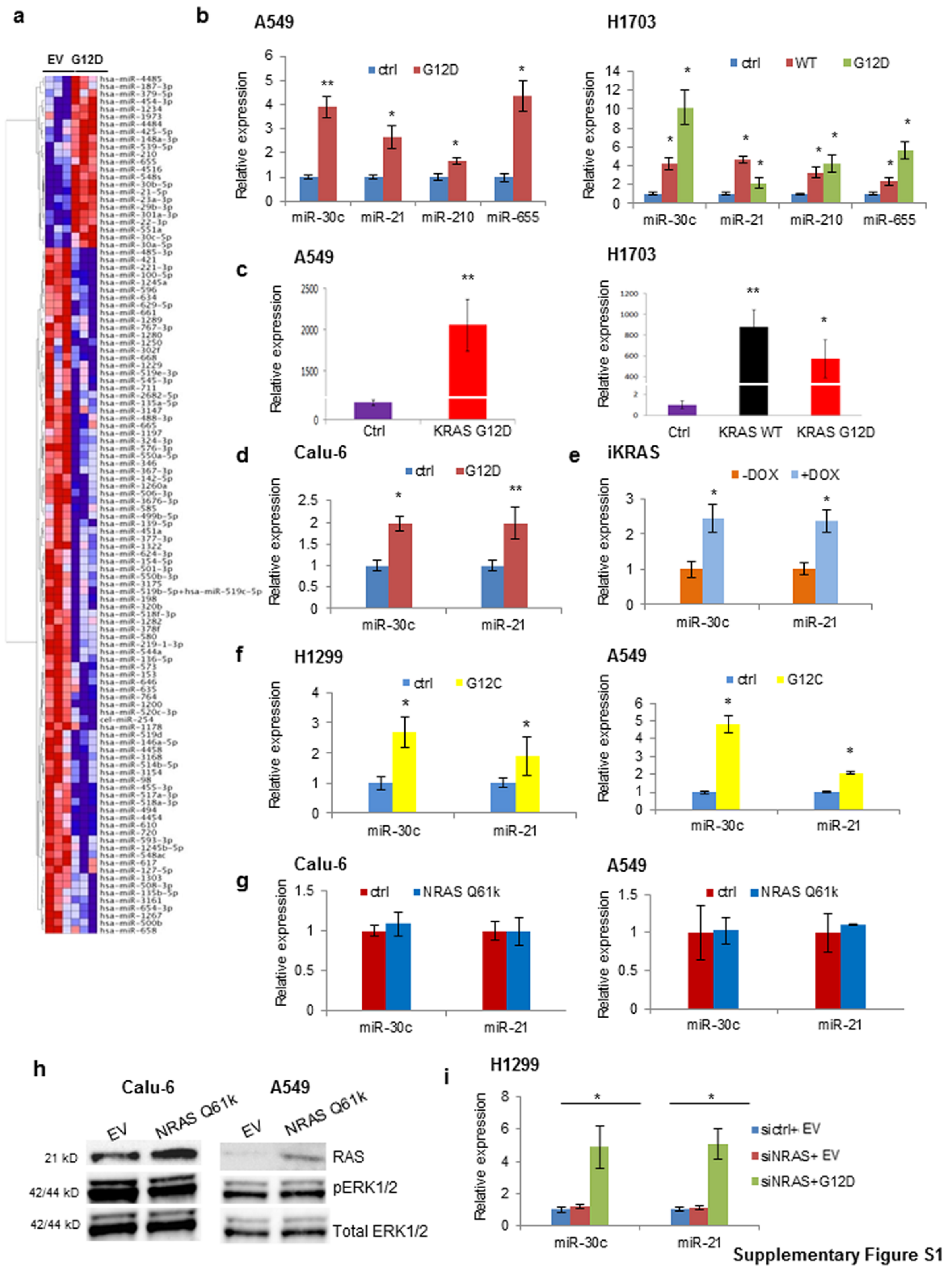

Supplementary Figure S1. **(a)** Complete miRNA heatmap of dysregulated miRNAs in H1299 cells overexpressing KRAS^{G12D} versus control cells ($P < 0.05$). **(b)** qPCR showing microRNA upregulation after KRAS^{WT} or KRAS^{G12D} overexpression in A549 and H1703 cells. **(c)** KRAS^{WT} and KRAS^{G12D} overexpression in A549 and H1703 cells. **(d)** MiR-30c and miR-21 upregulation in Calu-6 cells after

KRAS^{G12D} enforced expression. **(e)** Incresed levels of miR-30c and miR-21 in a KRAS inducible mouse cell line 48h after doxacyclin treatment. **(f)** Mutant KRAS (G12C) induces upregulation of miR-30c and mir-21 in H1299 and A549 cells. **(g-i)** Mutant NRAS does not affect ERK phosphorylation and miR-30c and miR-21 expression level. Bars indicate mean ± SD (*n* = 3). (**P* < 0.05, ***P* < 0.001) by two-tailed Student *t* test.

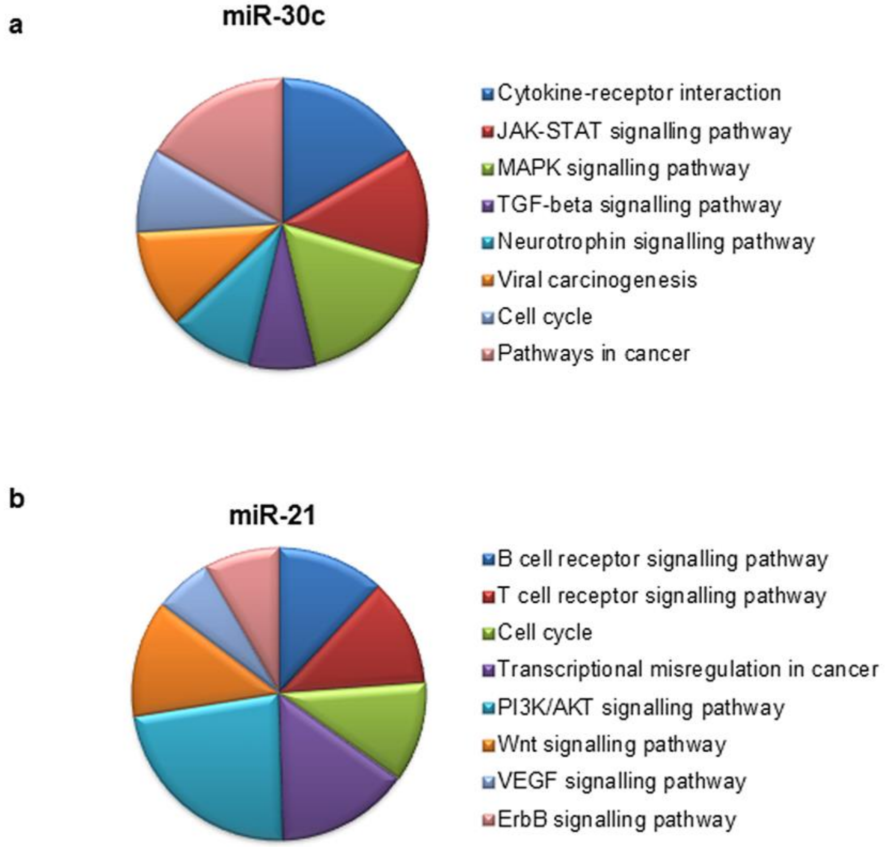

Supplementary Figure S2

Supplementary Figure S2. MiR-30c- and miR-21-modulated pathways. (a-b) DIANA miRPath v3.0 software was employed to analyze miR-30c- or miR-21-modulated signaling pathways and number of target genes based on TarBase v7.0 dataset.

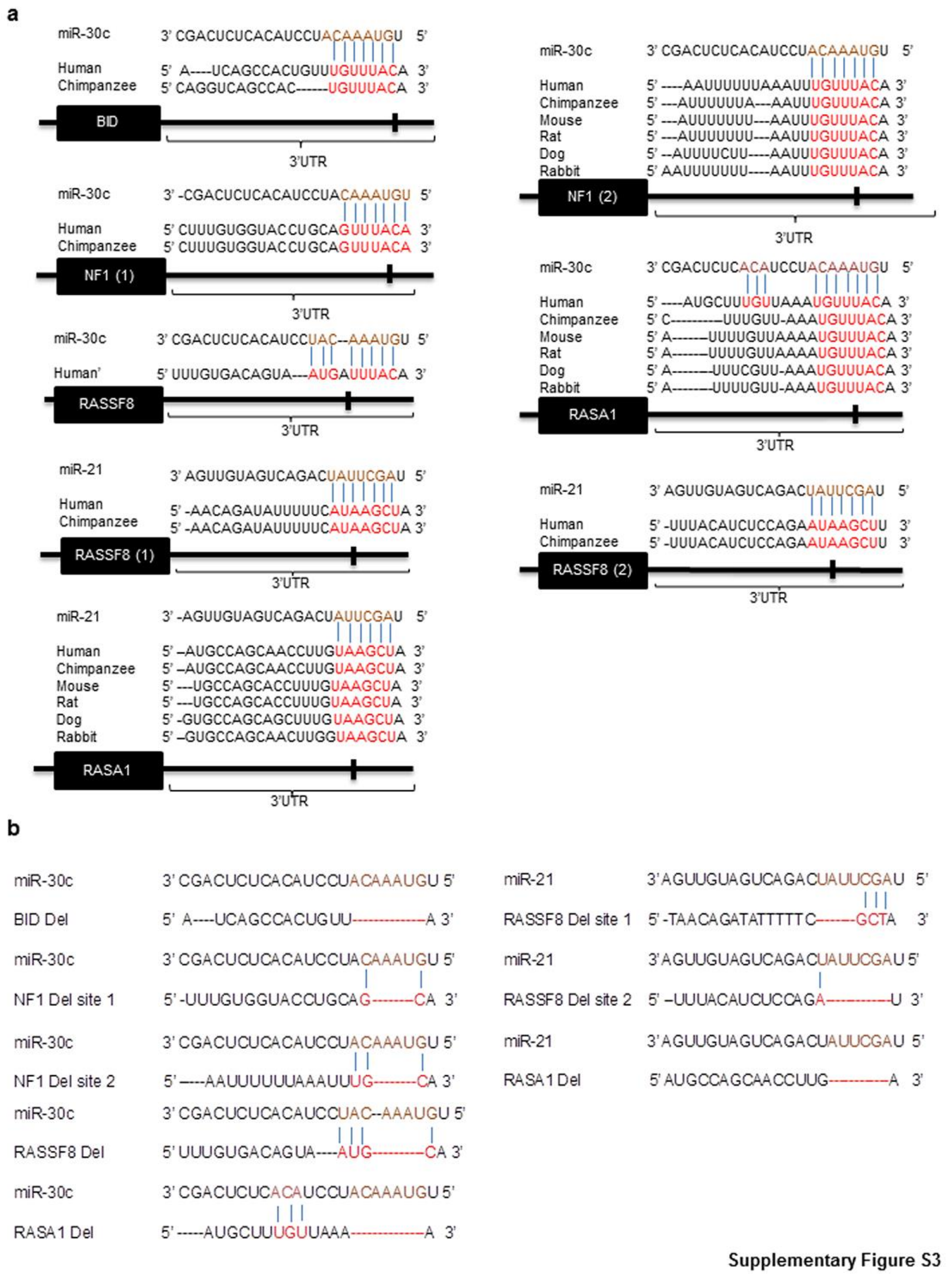

Supplementary Figure S3. MiRNA seed sequences in the 3' UTRs of target genes. (a) NF1, BID, RASSF8 and RASA1 present one or two miR-30c binding sites (NF1 nucleotides 1334-1340 and 1702-1709; BID nucleotides 724-731; RASSF8 nucleotides 1836-1843; RASA1 nucleotides 700-707). RASA1

and RASSF8 present one or two miR-21 binding sites, respectively (RASA1 nucleotides 179-185; RASSF8 nucleotides 690-697 and 3550-3556). **(b)** Deletions of miR-30c and miR-21 seed-matching sites in the 3'UTRs of target genes. In the figure is shown the alignment of the seed regions of miRNAs with the respective target 3' UTR. The sites of target mutagenesis are indicated in red –.

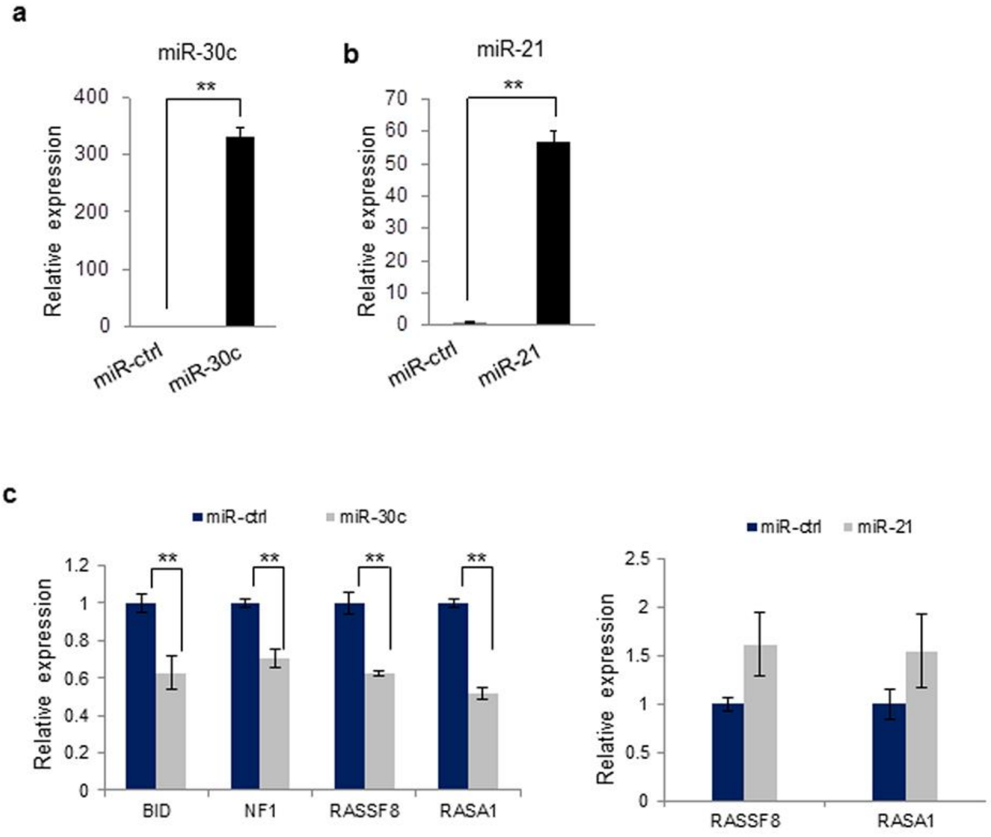

Supplementary Figure S4

Supplementary Figure S4. NF1, RASA1, RASSF8 and BID mRNA level after miR-30c and miR-21 overexpression. **(a-b)** Enforced expression of miR-30c and miR-21 in H1299 cells. **(c)** MiR-30c regulates BID, NF1, RASSF8 and RASA1 at the mRNA level. Bars indicate mean ± SD (n = 3). (*P < 0.05, **P < 0.001) by two-tailed Student t test.

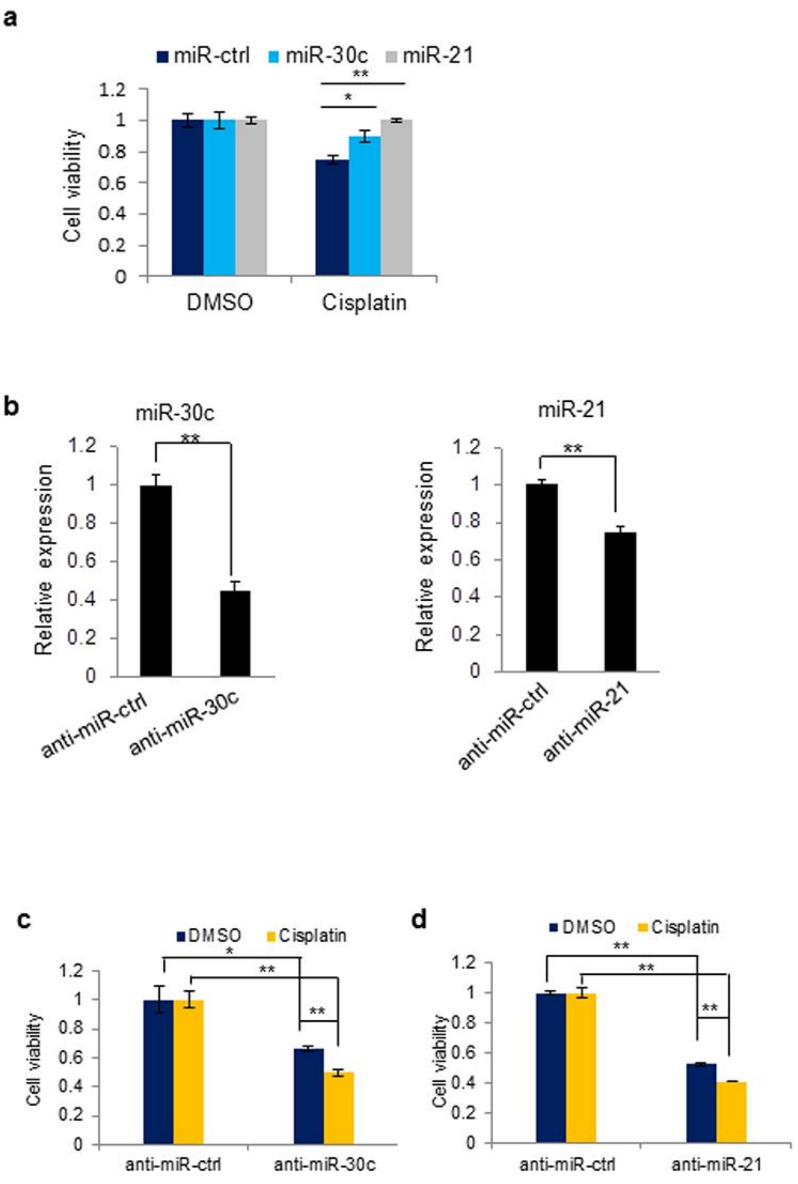

Supplementary Figure S5

Supplementary Figure S5. **Effect of miR-30c and miR-21 on cell proliferation and drug response. (a)** miR-30c and miR-21 overexpression reduced response to cisplatin of H292 cells. **(b)** qPCR of miR-30c and miR-21 in H1299 cells transfected with 100 nM of anti-miR-30c or anti-miR-21. **(c-d)** MiR-30c and miR-21 knockdown decreased cell proliferation and improved the response of A549 cells to cisplatin. Bars indicate mean ± SD (n = 3). (*P < 0.05, **P < 0.001) by two-tailed Student t test.

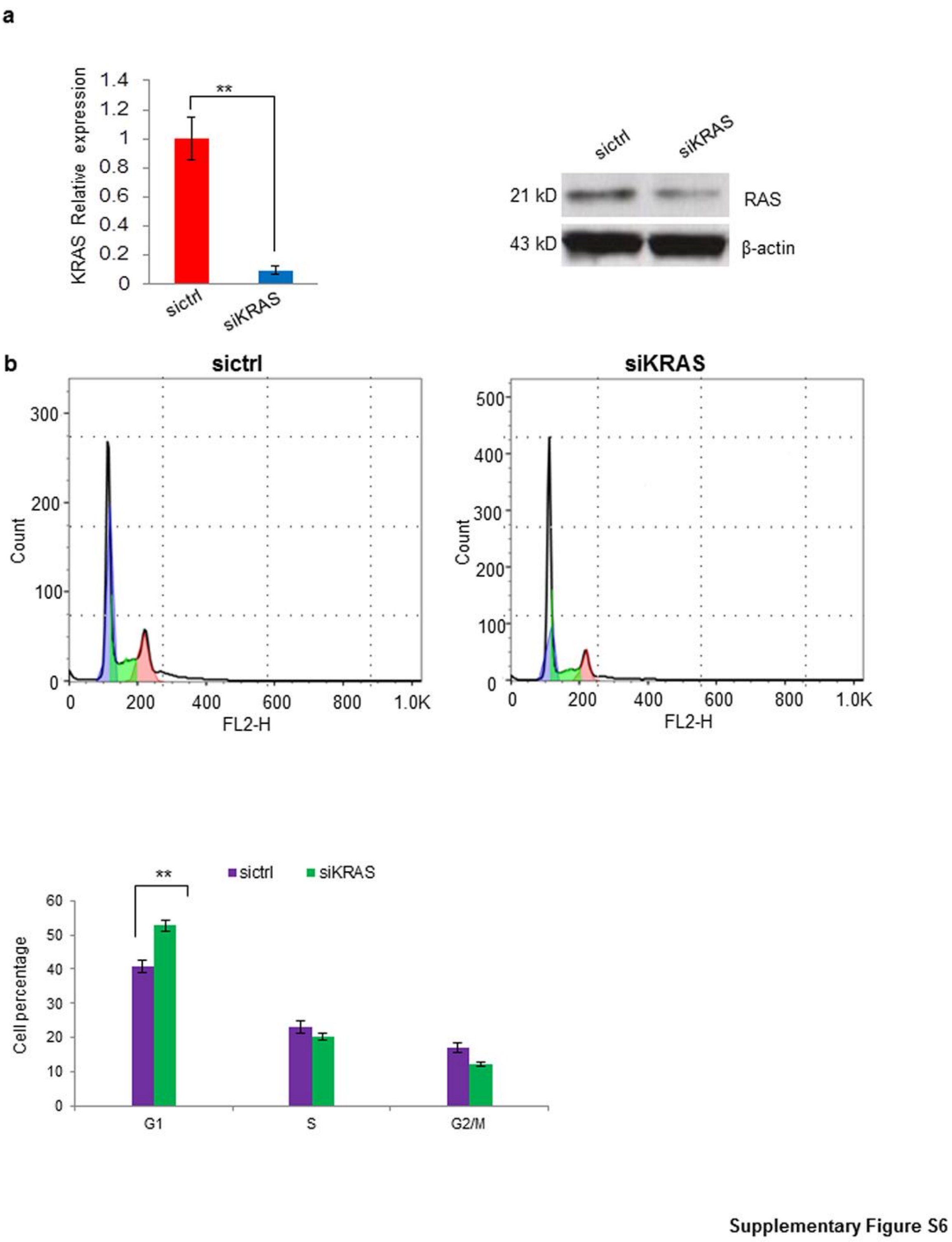

Supplementary Figure S6. **Effect of KRAS silencing on cell cycle. (a-b)** KRAS silencing in H1299 cells halts the cell cycle in G1 phase. Bars indicate mean ± SD (n = 3). (**P < 0.001) by two-tailed Student t test.

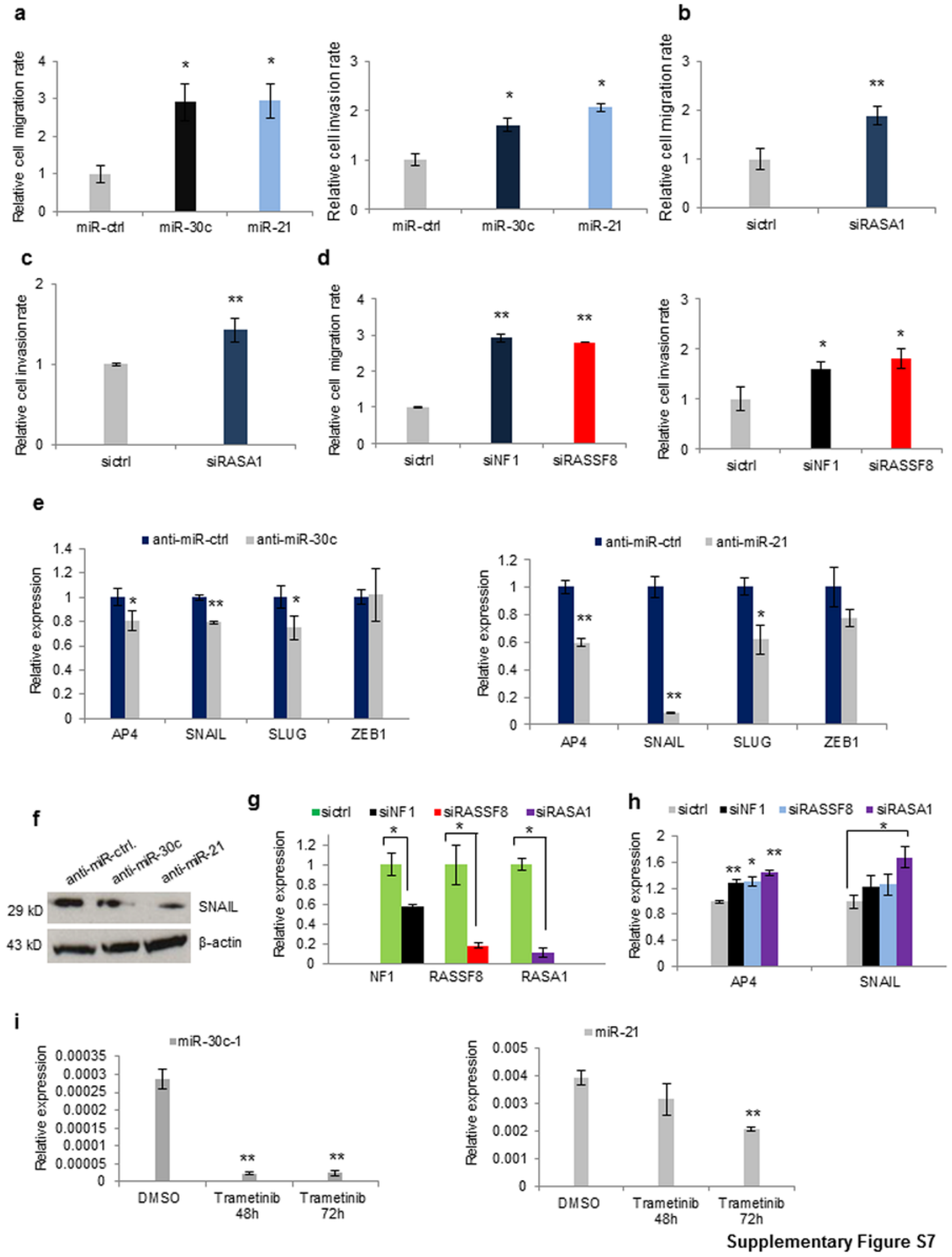

Supplementary Figure S7. MiR-30c and miR-21 promote migration and invasion via RASA1, NF1 and RASSF8. (a) Transient transfection of miR-30c and miR-21 in H1299 cells increased migration and invasion. **(b-d)** RASA1, NF1 and RASSF8 silencing promoted migration and invasion. **(e)** MiR-30c and miR-21 silencing repressed mesenchymal markers. **(f)** MiR-30c and miR-21 knock down reduced SNAIL

endogenous levels. **(g-h)** NF1, RASSF8 and RASA1 silencing increased AP4 and SNAIL mRNA levels. **(i)** miR-30c-1 and miR-21 precursor levels decrease after treatment of A549 cells with the MEK inhibitor Trametinib. Bars indicate mean ± SD (n = 3) and the P values were addressed by two-tailed Student t (*P < 0.05, **P < 0.001).

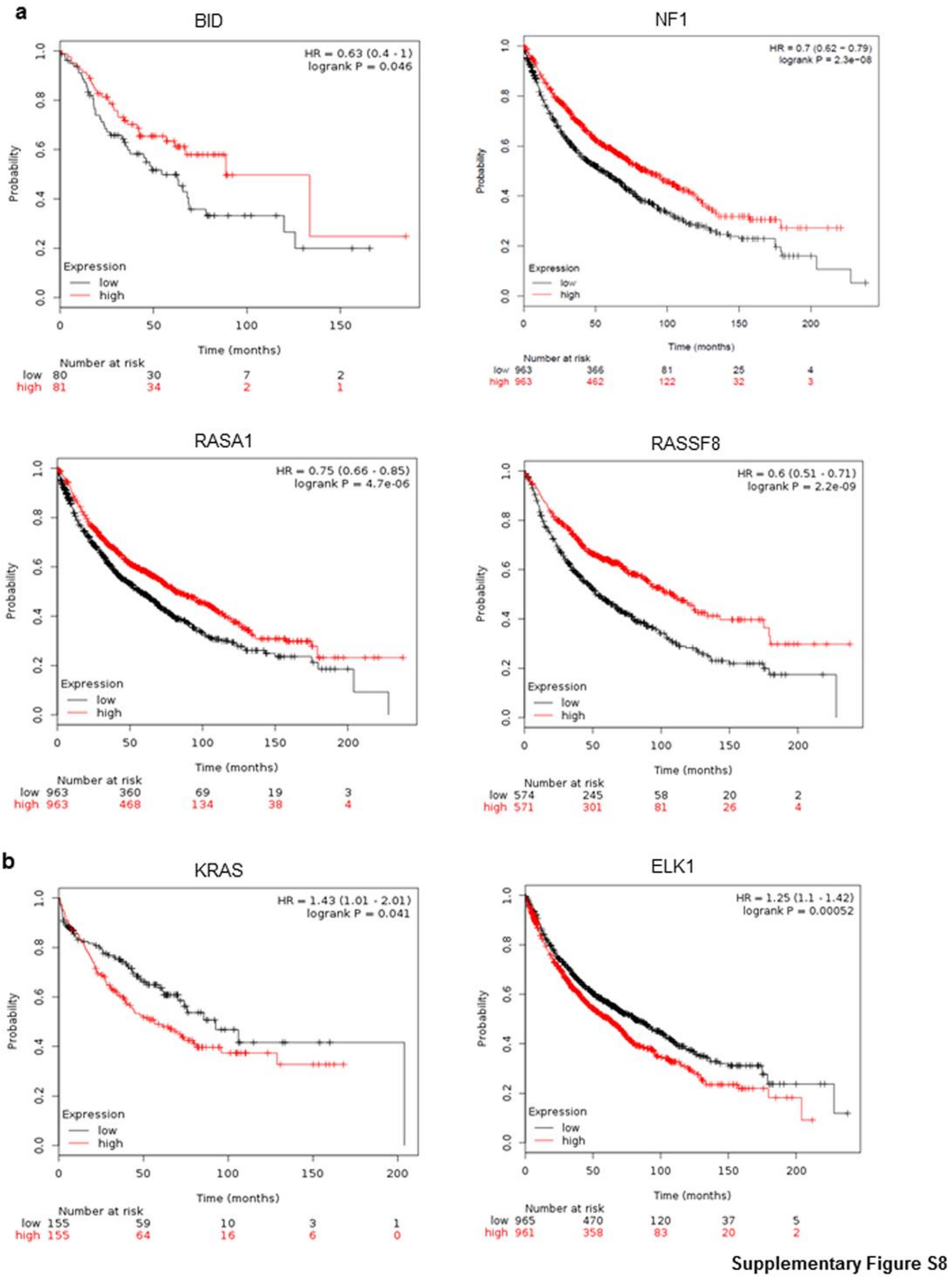

Supplementary Figure S8. High expression of KRAS and ELK1 and low expression of BID, NF1, RASSF8 and RASA1 predict poor lung adenocarcinoma patient outcome. (a) Kaplan-Meier Plotter showing that high expression of BID, NF1, RASSF8 and RASA1 correlates with longer survival NSCLC patients. HR=hazard ratio. **(b)** Conversely, high KRAS and ELK1 expression correlates with a shorter lifespan. HR=hazard ratio.

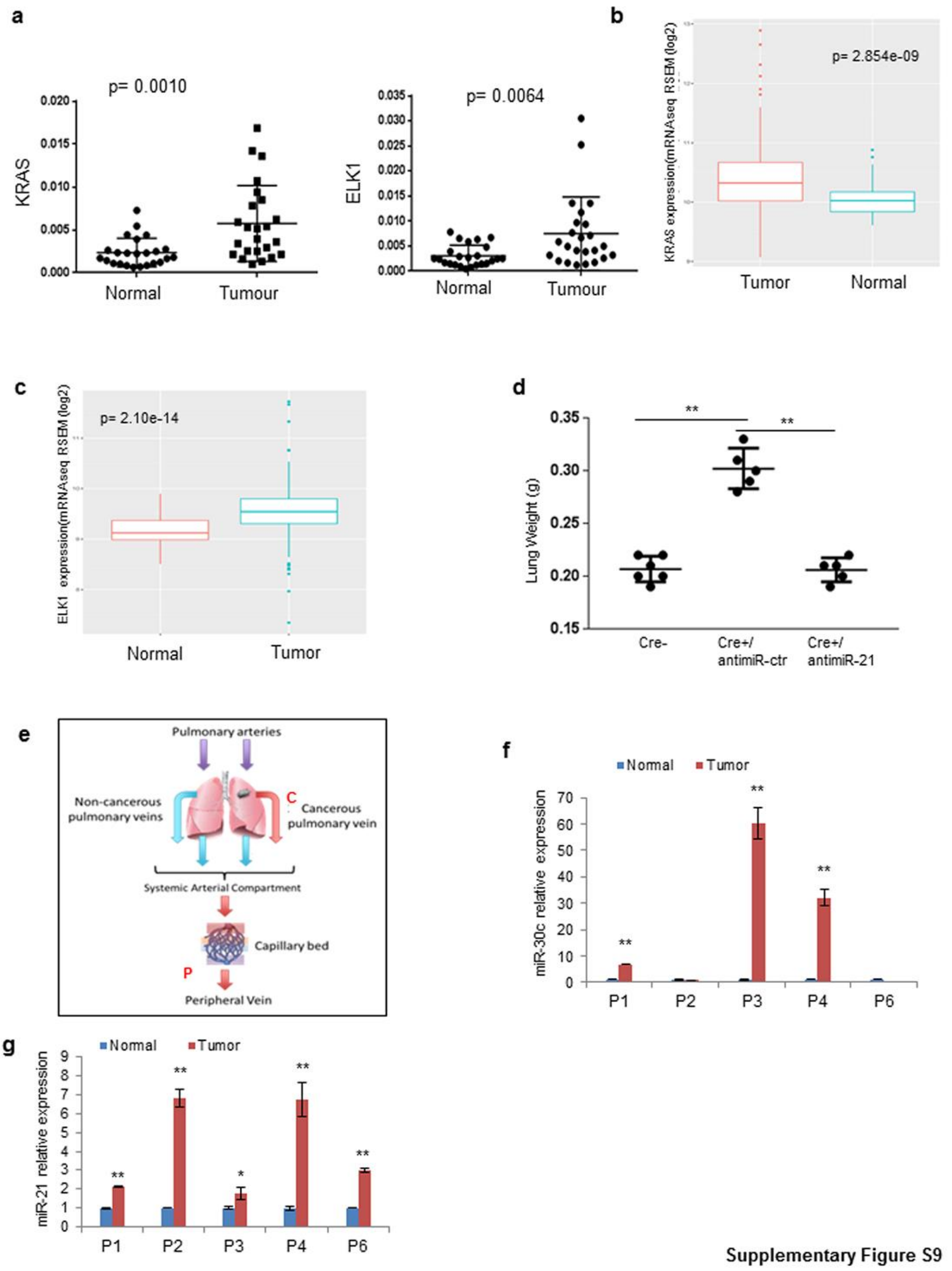

Supplementary Figure S9. MiR-30c and miR-21 in mouse and human specimens. **(a)** KRAS and ELK1 are upregulated in lung tumors compared to the normal counterpart (normal samples n=21; tumor sample n=21). **(b)** KRAS^{WT} expression in 155 adenocarcinoma samples (TCGA) compared to normal lung (n=46) **(c)** ELK1 expression in 513 adenocarcinoma samples (TCGA) compared to normal lung (n=46).

(d) Weight of lungs from KRAS^{G12D} mice treated with anti-miR-ctrl or anti-miR-21. **(e)** Schematic representation of sites of blood sampling. **C**=Pulmonary vein draining the cancer-bearing lobe, **P**=Peripheral vein. **(f-g)** MiR-30c and miR-21 expression in normal/matched tumor samples from the same patient as in Figure 6j-k. Normal/matched tumor samples were available in five out of six patients. P1-P6 = patient 1-6. Bars indicate mean ± SD (n = 3) and the P values were addressed by two-tailed Student t (*P < 0.05, **P < 0.001).

Supplementary Table S1

MiR-30c-modulated pathways

KEGG Pathway	p-value	Number of target genes	Gene names
Ubiquitin mediated proteolysis	5.768415e-09	21	UBE2R2, TRIM37, WWP1, CUL2, NEDD4L, HERC3, UBE2J1, UBE2I, SOCS3, CBLB, SKP2, SOCS1, UBE3C, BIRC6, RCHY1, NEDD4, UBE2F, UBE2D2, UBE2G1, PPIL2
Axon guidance	2.999473e-08	21	PLXNA2, EPHB2, EFNA3, KRAS, FYN, SEMA6B, PPP3CA, RASA1, NFAT5, PPP3CB, SRGAP3, NCK2, DPYSL2, NFATC2, UNC5C, CFL2, SEMA3A, PLXNC1, SEMA6D, NFATC3, ABL1
B cell receptor signalling pathway	1.146425e-07	13	BCL10, KRAS, PPP3CA, NFAT5, PIK3CD, PPP3CB, NFATC2, SOS1, DAPP1, RASGRP3, VAV3, NFATC3, LYN
Arrhythmogenic right ventricular cardiomyopathy	3.395797e-05	11	ITGA9, ITGA8, DMD, ACTN1, DSG2, GJA1, ITGA4, ATP2A2, CACNB2, ITGA6, SGCB
Amyotrophic lateral sclerosis	3.643886e-05	9	CAT, GRIA2, PPP3CA, CASP3, PPP3CB, GRIN2A, NEFM, SLCIA2, MAP3K5
Long-term protentiation	0.0001742297	10	CAMK2D, CAMK4, KRAS, GRIA2, PPP3CA, PPP1R12A, PPP3CB, GNAQ, GRIN2A, PAP1B
Protein processing in endoplasmic reticulum	0.0002949862	17	RAD23B, YOD1, SEC23A, UBE2J1, STT3B, SEC61A2, EDEM3, MANIA2, TUSC3, MAPK8, SEC24A, SSR3, UBE2D1, UBE2D2, UBE2G1, MARCH6, MAP3K5
T cell receptor signalling pathway	0.0003114126	13	BCL10, KRAS, FYN, PPP3CA, CBLB, NFAT5, PIK3CD, PPP3CB, NCK2, NFATC2, SOS1, VAV3, NFATC3
Mucin type O-Glycan biosynthesis	0.000315481	4	GALNT7, GALNT1, GALNT3, GALNT2
Hypertrophic cardiomyopathy	0.000315481	11	ITGA9, ITGA8, TTN, TPM4, DMD, ACTC1, ITGA4, ATP2A2. CACNB2, ITGA6, SGCB
Natural killer cell mediated cytotoxicity	0.0009460369	15	IFNGR2, KRAS, FYN, MICB, IFNAR2, PPP3CA, NFAT5, CASP3, PIK3CD, PPP3CB, NFATC2, SOS1, TNFRSF10B, VAV3, NFATC3
Dopaminergic synapse	0.0009460369	14	CAMK2D, PPP2R2B, GRIA2, PPP3CA, GNG10, SLC6A3, PPP3CB, MAPK8, SCN1A, GNAQ, PPP2R1B, GRIN2A, KCNJ3, CLOCK
Dilated cardiomyopathy	0.0009460369	11	ITGA9, ITGA8, TTN, TPM4, DMD, ACTC1, ITGA4, ATP2A2, CACNB2, ITGA6, SGCB
Gap junction	0.001038707	8	GUCY1A3, KRAS, SOS1, GNAQ, PDGFC, GJA1, MAP3K2, PDGFA
Regulation of actin cytoskeleton	0.001919905	19	ITGA9, APC, ITGA8, GNA13, FGF20, ARHGEF6, KRAS, PPP1R12A, PIK3CD, PFN2, ACTN1, PIP4K2B, CFL2, SOS1, PDGFC, ITGA4, ITGA6, VAV3, PDGFA
Neurotrophin signalling pathway	0.00202903	13	CAMK2D, SH2B3, CAMK4, KRAS, PIK3CD, MAPK8, KIDINS220, SOS1, IRS1, FOXO3, PAP1B,

			ABL1, MAPSK5
Glycosaminogycan biosynthesis-keratan sulphate	0.003637019	3	B4GALT4, CHST2, CHST1
Type II diabetes mellitus	0.003650644	6	IRS2, SOCS3, PIK3CD, SOCS1, MAPK8, IRS1
Cell cycle	0.004856012	12	SMAD2, CDC14A, ORC2, DBF4, STAG2, CCNA1, CCNE2, SKP2, YWHAZ, TFDP1, ABL1
Osteoclast differentiation	0.004966395	13	CAMK4, IFNGR2, CALCR, FYN, IFNAR2, SOCS3, PPP3CA, PIK3CD, PPP3CB, SOCS1, NFATC2, MAPK8, FOSL2
Long-term depression	0.005990866	8	GUCY1A3, GNA13, IGF1R, KRAS, GRIA2, GNAQ, PPP2R1B, LYN
Transcriptional misregulation in cancer	0.01094325	16	BMI1, CCNT2, RUNX1, ERG, IGF1R, RUNX2, CCNA1, JMJD1C, DDX5, ATF1, BCL6, KMT2A, PER2, HOXA11, PAX3, PDGFA
PI3K/AKT signaling pathway	0.01423164	25	PRLR, ITGA9, ITGA8, EFNA3, PPP2R2B, IGF1R, FGF20, KRAS, IFNAR2, GNG10, DDIT4, PIK3CD, EIF4E, CCNE2, SOS1, IL2RA, YWHAZ, IRS1, PDGFC, FOXO3, ITGA4, ITGA6, PPP2R1B, BCL2L11, PDGFA
Pancreatic secretion	0.02022249	10	RAB8A, RAB3D, ATP2B1, PLA2G12A, RAB27B, RAB11A, GNAQ, ATP2A2, RAP1B, PLA2G2C
Wnt signaling pathway	0.02691257	14	CAMK2D, CSNK2A2, LRP6, SMAD2, APC, PPP3CA, NFAT5, PPP3CB, NFATC2, MAPK8, SCNK1A1, NFATC3, PPP2R1B, TBL1XR1
VEGF signaling pathway	0.02705103	7	KRAS, PPP3CA, NFAT5, PIK3CD, PPP3CB, NFATC2, NFATC3
Amphetamine addiction	0.02705103	8	CAMK2D, CAMK4, SIRT1, GRIA2, PPP3CA, SLC6A3, PPP3CB, GRIN2A
N-Glycan biosynthesis	0.02708313	6	DOLPP1, STT3B, ALG9, MAN1A2, ALG10B, TUSC3
Viral myocarditis	0.04341414	7	FYN, DMD, CASP3, MYH11, ABL2, SGCB, ABL1
ErbB signaling pathway	0.04838723	9	CAMK2D, KRAS, CBLB, PIK3CD, NCK2, MAPK8, SOS1, ABL2, ABL1

MiR-21-modulated pathways

KEGG Pathway	p-value	Number of target genes	Gene names
Cytokine-receptor interaction	6.116134e-07	9	CNTFR, FASLG, LIFR, CCL20, ACVR2A, CCL1, TGFBR2, BMPR2, IL6L
Steroid biosynthesis	0.001228031	2	SC5D, SOAT1
JAK-STAT signaling pathway	0.001256332	7	STAT3, CNTFR, LIFR, PIK3R1, SPRY1, SPRY2, IL6R
MAPK signaling pathway	0.001994296	9	NTF3, RASA2, FASLG, RASGRP1, DUSP8, GFG18, RPS6KA3, MEF2C, TGFBR2
TGF-beta signaling pathway	0.005704367	4	ACVR2A, SMAD7, TGFBR2, BMPR2
Pancreatic cancer	0.006055022	4	STAT3, E2F3, PIK3R1, TGFBR2
N-Glycan biosynthesis	0.007484419	2	MAN1A2, ST6GAL1
Hepatitis B	0.01027284	5	STAT3, CREB5, FALSG, E2F3, PIK3R1
Neurotrophin signaling pathway	0.01451633	5	NTF3, FRS2, FASLG, PIK3R1, PRS6KA3
Viral carcinogenesis	0.01451633	6	STAT3, RASA2, CREB5, SKP2, PIK3R1, RBPJ
Small cell lung cancer	0.01451633	4	SKP2, E2F3, PIK3R1, COL4A1
Regulation of actin cytoskeleton	0.02511277	6	TIAM1, VCL, PIKFYVE, PIK3R1, FGF18, ARHGEF7
Cell cycle	0.02679686	5	STAG2, WEE2, SKP2, E2F3, CDC25A
Pathways in cancer	0.02940469	9	STAT3, FASLG, SKP2, E2F3, PIK3R1, FGF18, TRGBR2, EGLN1, COL4A1

Supplementary Table S2

Primers used to amplify 3'UTRs of miR-30c and miR-21 target genes

Gene	Primer	Sequence
**miR-30c targets**		

BID	Forward	5' CGC TCT AGA GCA CTC AAA TGA TGG GAA GTC 3'
	Reverse	5' GCG TCT AGA CAT AGC TTA CCA CTG GAA CAG 3'
NF1 (1)	Forward	5' ACT TCT AGA CTG GTC AGC CTG CAT TAG TAT GAC AG TAG G 3'
	Reverse	5' ACT TCT AGA GAA ACA GCA CTA CAG GAA ACT CCC CTC 3'
NF1 (2)	Forward	5' AGC TCT AGA GAG GGG AGT TTC CTG TAG TG 3'
	Reverse	5' ATA TCT AGA CAC TGG GTT GTG GGA GAG CC 3'
RASSF8	Forward	5' GGC TCT AGA CTT TGG GTT CTT GGA GTT ATC A 3'
	Reverse	5' GGA TCT AGA GTT TTT CGT GGT ATG CCA AGG 3'
RASA1	Forward	5' ACG TCTAGA GTAACTACTTCCTGATTAGG 3'
	Reverse	5' CGC TCTAGA AGGTTGTCTGCATAATC 3'
**miR-21 targets**		
RASSF8 (1)	Forward	5' AGC TCT AGA GAA ACT GTC CTA GCC ATT GC 3'
	Reverse	5' GAC TCT AGA CAA CCC AGC TTT CAG GTA TC 3'
RASSF8 (2)	Forward	5' ACT TCT AGA GCT GTG TTC ACA TCC CTC TC 3'
	Reverse	5' GCG TCT AGA TGC TCA CAA TTA GTG AAC ATG G 3'
RASA1	Forward	5' GCG TCT AGA GTG ATG TGT GAG CTA TGC AA 3'
	Reverse	5' GCG TCT AGA ACA GTA AGA ACA GTC TGC AAT C 3'

Primers used to delete miR-30c and miR-21 binding sites in target genes

Gene	Primer	Sequence
**miR-30c**		
BID Del	Forward	5' GGCCCAGGTCAGCCACTGTTAAAATCAGGTAACTAACT 3'
	Reverse	5' AGTTAGTTACCTGATTTTAACAGTGGCTGACCTGGGCC 3'
NF1 (1)	Forward	5' TTCTTTGTGGTACCTGCAGCAAAATAATTTGACTTCAGTGAGC 3'
	Reverse	5' GCTCACTGAAGTCAAATTATTTTGCTGCAGGTACCACAAAGAA 3'
NF1 (2)	Forward	5' GAATTTAATTTTTTAAATTTGCAGTCCTGGGAAAAGTAAG 3'
	Reverse	5' CTTACTTTTCCCAGGACTGCAAATTTAAAAAATTAAATTC 3'
RASSF8	Forward	5' ATAGATTTTGTGACAGTAATGCATATGCCCAATATATGCCTTA 3'
	Reverse	5' TAAGGCATATATTGGGCATATGCATTACTGTCACAAAATCTAT 3'
RASA1	Forward	5' GATCTCATAATGCTTTGTTAAATGCAAGTAAATAGTTTGA 3'
	Reverse	5' TCAAACTATTTACTTGCATTTAACAAAGCATTATGAGATC 3'
**miR-21**		
RASSF8 (1)	Forward	5' GCATAACAGATATTTTTCGCTAAATTGTATGTATAAAAC
	Reverse	5' GTTTTATACATACAATTTAGCGAAAAATATCTGTTATGC 3'
RASSF8 (2)	Forward	5' GCTGCTTTACATCTCCAGATTCGATGCCAGGACAGTG 3'
	Reverse	5' CACTGTCCTGGCATCGAATCTGGAGATGTAAAGCAGC 3'
RASA1	Forward	5' CTATGCCAGCAACCTTGATCTGTGCAGGATATTTGCAC 3'
	Reverse	5' GTGCAAATATCCTGCACAGATCAAGGTTGCTGGCATAG 3'

Supplementary Table S3

Primers used to amplify miR-30c and miR-21 promoter regions

MicroRNA	Primer	Sequence

miR-30c-(1)	Forward	5’ GGTACCATGACGCTGACCAGAGAGGATTCACAAGA 3’
	Reverse	5’ AGATCTATTCAGTGAGTGAGTAGCTGAGGGGAGAGG 3’
miR-30c-(2)	Forward	5’ GGTACCTTATTACTGTGTATCCCATCATTAAGG 3’
	Reverse	5’ AGATCTGTCACAGGAAAAGATCAGAAAAGTATAC 3’
miR-21	Forward	5’ ACTGGTACC AGTTATGCCAAACGAATCCAG 3’
	Reverse	5’ TATCTCGAG CGCTCAAGAAAACGGACAC 3’

Primers used to delete ELK1 binding site(s) in miR-30c and miR-21 promoter regions

MicroRNA	Primer	Sequence
miR-30c Del (1)	Forward	5’ CTCTTTCCTTCTCCACTTCTATTCTTTCCCTCC 3’
	Reverse	5’ GCAGGAGGGAAAGAATAAGAAGTGGAGAAG 3’
miR-30c Del (2) A	Forward	5’ ATTATGTACTGTGAAGGAGTTTGGCGAAAT 3’
	Reverse	5’ TATTCGCCAAACTCCTTCACAGTACATAATC 3’
miR-30c Del (2) B	Forward	5’ ATCTTAGGATGTGCAAGAAGAAAACAAGAAC 3’
	Reverse	5’ CAGTTCTTGTTTTCTTCTTGCACATCCTAAG 3’
miR-21 Del (1)	Forward	5’ GCCTTAAATTGGGAGGACTCCAAGAAAATTCCCTTTTCCAACCTG 3’
	Reverse	5’ CAGGTTGGAAAAGGGAATTTTCTTGGAGTCCTCCCAATTTAAGGC 3’
miR-21 Del (2)	Forward	5’ ATATTCCATCCTGCCCAACCCCATCCTCAAAAAAGGGCCAT 3’
	Reverse	5’ ATGGCCCTTTTTTGAGGATGGGGTTGGGCAGGATGGAATAT 3’

Supplementary Table S4 Primers used for ChIP

MicroRNA	Primer	Sequence
miR-30c-ELK1(1)	Forward	5’ GGA TTA TGT CT GTT TAA GG AAG 3’
	Reverse	5’ CCT GCT CAG TTC TTG TTT TCT TC 3’
miR-30c-ELK1(2)	Forward	5’ GGATTATGTACTGTTTAAGGAAGG 3’
	Reverse	5’ CCT GCT CAG TTC TTG TTT TCT TC 3’
miR-21-ELK1(1)	Forward	5’ GTTGAACTCAGAGGAGAAAACT 3’
	Reverse	5’ TGTCAGTGCAAAGTATGGAC 3’
miR-21-ELK1 (2)	Forward	5’ GATTGCTTTGTTTTCCTAAGTCATC 3’
	Reverse	5’ TAAGACACCAACCAGATTTTCCTTATAC 3’

Supplementary Table S5 Primers used for qPCR

Gene	Primer	Sequence
AP4	Forward	5’ GCAGGCAATCCAGCACAT 3’
	Reverse	5’ GGAGGCGGTGTCAGAGGT 3’
SNAIL	Forward	5’ GCACATCCGAAGCCACAC 3’
	Reverse	5’ GGAGAAGGTCCGAGCACA 3’
SLUG	Forward	5’ TGGTTGCTTCAAGGACACAT 3’
	Reverse	5’ GTTGCAGTGAGGGCAAGAA 3’
ZEB1	Forward	5’ TCAAAAGGAAGTCAATGGACAA 3’
	Reverse	5’ GTGCAGGAGGGACCTCTTTA 3’
CDH1	Forward	5’ CCCGGGACAACGTTTATTAC 3’
	Reverse	5’ GCTGGCTCAAGTCAAAGTCC 3’
β-actin	Forward	5’ TGACATTAAGGAGAAGCTGTGCTAC 3’
	Reverse	5’ GAGTTGAAGGTAGTTTCGTGGATG 3’

Supplementary Table S6. Antibodies used for Western Blot, IF and ChIP

Antibody	Company	Application	Dilution
BID	Cell signalling	WB	1:1000
NF1	Bethyl Laboratories	WB<br>IF	1:1000<br>1:100
RASSF8	Abcam	WB<br>IF	1:1000<br>1:100
RASA1	Abcam	WB<br>IF	1:1000<br>1:100
RAS	Cell signalling	WB	1:1000
Erk1/2	Promega	WB	1:1000
pErk1/2	Entrez-Gene	WB	1:1000
ELK1	Cell signalling	WB	1:1000
AKT	Cell signalling	WB	1:1000
pAKT	Cell signalling	WB	1:1000
IκB-α	Santa Cruz	WB	1:1000
β-actin	Santa Cruz	WB	1:1000
α-Tubulin	Cell signalling	WB	1:1000
ELK1	Abcam	Chip	1:50
Lamin B1	Cell signalling	WB	1:1000
